# Clinical outcomes-dependent IgG epitope profiling in HTLV-1 reveals differential recognition of pathogen-derived antigens

**DOI:** 10.3389/fimmu.2026.1755133

**Published:** 2026-02-25

**Authors:** Natali Espasiani Cilento, João Vitor da Silva Borges, Nicolle Rakanidis Machado, Lais Alves do Nascimento, Anna Luisa Baratelli Moreira, Lhays Ozório Passos, Aline Boveto Santamarina, Jorge Casseb, Sabri Saeed Sanabani, Jefferson Russo Victor

**Affiliations:** 1 Post Graduation Program in Health Sciences, Santo Amaro University (UNISA), São Paulo, Brazil; 2 School of Medicine, Santo Amaro University (UNISA), São Paulo, Brazil; 3 Laboratory of Medical Investigation LIM-56, Division of Dermatology, Medical School, University of São Paulo, São Paulo, Brazil; 4 Laboratory of Medical Investigation LIM-03, Clinics Hospital, University of Sao Paulo, Medical School, Sao Paulo, Brazil

**Keywords:** antibody repertoire, ATLL, autoantibodies, cross-reactivity, HAM/TSP, HTLV-1, IgG, immune modulation

## Abstract

Human T-lymphotropic virus type 1 (HTLV-1) infection presents a wide clinical spectrum ranging from lifelong asymptomatic carriage to severe inflammatory neurodegeneration (HAM/TSP) or adult T-cell leukemia/lymphoma (ATLL). Although IgG responses contribute to viral control and immunopathology, the extent to which HTLV-1 clinical outcomes shape pathogen-derived IgG repertoires remains unclear. In this study, we applied a high-density infectious-disease epitope microarray containing 4,345 linear epitopes from viral, bacterial, parasitic, and fungal pathogens to profile IgG responses in healthy controls (HCs), asymptomatic carriers (ACs), HAM/TSP patients, and ATLL patients. Signal intensities were quantified in arbitrary units, and recognized epitopes were evaluated using similarity clustering (80% identity threshold) to assess repertoire structure. HTLV-1–infected individuals exhibited extensive remodeling of humoral immunity, with marked differences in the breadth and intensity of IgG recognition across clinical groups. HAM/TSP patients displayed broad and high-magnitude responses consistent with chronic inflammation and heightened Th1 activation, whereas ATLL patients recognized the largest number of epitopes but with distinct patterns indicative of altered B-cell regulation. Enhanced IgG responses to *Mycobacterium tuberculosis*, *Strongyloides stercoralis*, *Toxoplasma gondii*, and *Plasmodium* species were consistent with known co-infection susceptibilities in HTLV-1. Epitope similarity analysis revealed hundreds of low-redundancy clusters across all groups, arguing against simple linear cross-reactivity and suggesting phenotype-specific reshaping of B-cell selection and idiotypic networks. These findings demonstrate that HTLV-1 infection produces distinct, clinically dependent IgG epitope signatures across multiple pathogen classes, with potential relevance for understanding HTLV-1 pathogenesis and informing future studies integrating epitope mapping with B-cell repertoire analysis.

## Introduction

Human T-lymphotropic virus type 1 (HTLV-1) is a retrovirus associated with several inflammatory conditions, including pulmonary alveolitis, chronic dermatitis, arthropathy, uveitis, conjunctivitis, and interstitial keratitis (1–5). Although the majority of infected individuals remain asymptomatic, approximately 1–5% progress to adult T-cell leukemia/lymphoma (ATLL) or HTLV-1–associated myelopathy/tropical spastic paraparesis (HAM/TSP) (6–9). Increasing attention has been devoted to understanding how naturally occurring and infection-driven IgG repertoires are shaped (10–12). Nevertheless, experimental evidence remains insufficient to fully elucidate the molecular principles underlying the “hooks without bait” hypothesis (13). This concept posits that IgG idiotypes arising from environmental exposures, genetic background, and infectious triggers form distinct antibody sets capable of interacting with conserved or clonally expressed molecules on lymphocytes and other immune cells, thereby modulating immune function. Depending on the idiotype–cell interaction, these effects may be regulatory or pro-inflammatory, underscoring the potential relevance of idiotypic networks to disease pathogenesis and therapeutic innovation.

A substantial body of work has demonstrated that discrete IgG idiotype repertoires can independently modulate immune responses in both murine and human lymphocytes (14–17). These influences include alterations in cytokine production by thymic and peripheral αβ T cells, γδ T cells, and B cells, with effects shaped by the immune status of the donor. For instance, IgG from atopic individuals modulates T-cell IFN-γ production (18), while IgG from patients with atopic dermatitis regulates IL-17, IL-22, and IL-10 secretion (19–21). IgG from non-atopic donors affects IL-17, IFN-γ, and IL-10 production (22, 23), and IgG from HIV-1–exposed uninfected and infected individuals can modulate IFN-γ responses in both T and B cells (24). In B cells, IgG from non-atopic individuals has been shown to induce IL-10–producing B cells (B10 cells) in infant thymus and adult PBMCs (25), supporting previous observations in murine allergy models (26, 27).

In HTLV-1 infection, effective control of viral replication depends on multiple effector mechanisms, including IgG antibody production (28). The magnitude and quality of the humoral response may influence clinical outcomes, helping to distinguish asymptomatic carriers from those who develop HAM/TSP or ATLL. Elevated HTLV-1–specific antibody titers have been associated with HAM/TSP (29), suggesting that the breadth or specificity of the IgG response may contribute to inflammatory disease development.

Recent work has shown that IgG from HAM/TSP patients increases IL-17 production by CD4^+^ T cells, decreases IL-4^+^ CD4^+^ T cells, enhances IFN-γ production by CD8^+^ T cells, and reduces IL-4^+^ CD8^+^ T cells. Conversely, IgG from ATLL patients decreases IL-4^+^ CD4^+^ T cells, reduces IFN-γ^+^ γδ T cells, and lowers IL-10^+^ B-cell frequencies. IgG from both groups reduces IFN-γ^+^ B cells (30). Another recent study suggests that HTLV-1 infection may promote the development of distinct IgG repertoires with broad reactivity toward human proteins, potentially contributing to immune dysregulation and offering a partial explanation for divergent clinical trajectories in HAM/TSP and ATLL (31). Together, these findings indicate a wide spectrum of IgG-mediated immune modulation in HTLV-1 infection and raise the possibility that self-protein recognition may occur, although the origins of these repertoires—particularly in the context of environmental exposure—remain unclear.

In the context of a distinct infectious disease, but using methodological approaches comparable to those previously applied in HTLV-1 research, studies in coronavirus disease 2019 (COVID-19) have demonstrated that infection with SARS-CoV-2 can substantially influence the IgG idiotype repertoire of infected individuals. SARS-CoV-2 infection has been shown to modulate IgG recognition profiles against a broad range of host proteins, including molecules associated with major peripheral immune cell populations such as T cells, B cells, and monocytes (32), as well as proteins expressed across multiple organ systems (33). Notably, these alterations have been reported to correlate with disease severity, underscoring the relevance of investigating infection-induced changes in IgG repertoires in relation to clinical status.

In addition, a recent study aligned with the conceptual framework of the present work evaluated the capacity of IgG idiotypes to recognize a broad panel of more than one hundred epitopes derived from several distinct pathogens (34). This study demonstrated that SARS-CoV-2 infection induces a severity-dependent reshaping of the IgG epitope repertoire that extends beyond viral antigens to include epitopes from unrelated pathogens. Collectively, these observations support the notion that infectious diseases may broadly remodel humoral immune recognition through idiotype-driven mechanisms and highlight the potential clinical relevance of IgG repertoire diversity and specificity.

In this context, HTLV-1 infection represents a particularly relevant model, as this retrovirus is associated with a spectrum of inflammatory and immune-mediated conditions whose underlying pathogenic mechanisms remain incompletely understood. Importantly, the distinct clinical phenotypes associated with HTLV-1 infection typically emerge or persist years after primary infection, suggesting that long-term immune modulation may play a critical role in disease development. Drawing parallels with observations from other infectious diseases, including COVID-19, two key questions arise: whether exposure to unrelated pathogens can influence clinical outcomes in HTLV-1–infected individuals, and whether HTLV-1 infection predisposes individuals to distinct patterns of IgG recognition against other pathogens, potentially affecting susceptibility and secondary clinical manifestations.

To begin addressing these hypotheses, the present study applies a high-throughput IgG epitope-profiling platform capable of interrogating 4,345 linear epitopes derived from a wide range of human pathogens—including viruses, bacteria, parasites, and fungi—to generate a comprehensive landscape of pathogen-associated IgG reactivity and corresponding epitope-recognition profiles in HTLV-1 infection.

## Methods

### Samples

Serum specimens were obtained from a subset of participants enrolled in a larger cohort comprising 233 HTLV-1–infected individuals. This subset included asymptomatic carriers (ACs; n = 14; 12 males, 2 females; mean age ± SE: 52.7 ± 2.7 years; median proviral load: 13 copies/1,000 PBMCs), patients diagnosed with HTLV-1–associated myelopathy/tropical spastic paraparesis (HAM/TSP; n = 16; 10 males, 6 females; age: 57.4 ± 2.2 years; median proviral load: 162 copies/1,000 PBMCs), and individuals with adult T-cell leukemia/lymphoma (ATLL; n = 11; 7 males, 4 females; age: 48.4 ± 4.6 years; median proviral load: 502 copies/1,000 PBMCs). All ATLL cases included in this study were classified as the leukemic subtype and had undergone antiviral therapy. No comorbid conditions known to directly affect the generation of specific IgG idiotypes were identified among the enrolled patients. Patients were recruited from the HTLV-1 outpatient services of the University of São Paulo and the Instituto de Infectologia “Emílio Ribas.” Healthy controls (HCs; n = 40; 17 males, 23 females; mean age ± SE: 47.5 ± 2.5 years) were confirmed seronegative for HTLV-1 at the time of sample collection.

HTLV-1 infection was initially screened using the Murex HTLV I + II (Abbott/Murex, Wiesbaden, Germany) and Vironostika HTLV I/II (bioMérieux bv, Boxtel, Netherlands) assays, with confirmatory testing performed using HTLV BLOT 2.4 (Genelabs Diagnostics, Singapore). HAM/TSP diagnoses followed WHO criteria for HTLV-1–associated neurological disease, while ATLL diagnoses required serologic evidence of HTLV-1 infection along with cytological or histopathological confirmation of T-cell malignancy.

### Infectious disease epitope microarray

IgG reactivity was assessed using the PEPperCHIP^®^ Infectious Disease Epitope Microarray (PEPperPRINT GmbH, Heidelberg, Germany). This platform contains 4,345 linear peptide epitopes derived from 53 viral, 25 bacterial, 23 parasitic, and 1 fungal species known to infect humans. The full list of epitopes, molecule of origin and microorganisms of origins presented on the array appears in **Supplementary Table S1** (Tab 1) including Immune Epitope Database (IEDB) accession numbers.

### Quality control and sample incubation

To assess nonspecific binding, each microarray was initially incubated with secondary and control antibodies only. This step enabled the determination of inherent background reactivity and ensured that subsequent peptide-level signals were not confounded by nonspecific fluorescence. For each individual analysis, the arbitrary unit (A.U.) value corresponding to background reactivity was subtracted from the signal obtained after incubation with serum samples. Negative values resulting from background subtraction were set to zero. The background-corrected values were considered representative of sample-specific IgG reactivity. Signals equal to 0 A.U. were classified as exhibiting *no detectable reactivity*, whereas signals greater than 0 A.U. were classified as *recognized epitopes*.

Human serum samples were individually diluted at 1:500, as determined by prior titration experiments optimizing the signal-to-noise ratio, and applied to the microarrays under standardized incubation conditions. Bound IgG was detected using a fluorescently labeled secondary antibody, and microarrays were scanned using an Innopsys InnoScan 710-IR system.

HA control peptides positioned along the microarray margins served as internal positive controls and were probed with an anti-HA antibody to verify assay consistency and slide integrity throughout the process.

### Image processing and fluorescence quantification

Scanning generated 16-bit grayscale TIFF files, chosen for their superior dynamic range relative to RGB images. Signal extraction followed an automated workflow that obtained raw, foreground, and background fluorescence values for each peptide. Median foreground intensities from duplicate spots were averaged, and intra-duplicate variation was monitored by calculating spot-to-spot deviation.

A deviation limit of 40% was imposed to ensure reproducibility; signals exceeding this threshold were excluded unless manual inspection justified retention. Intra-array reproducibility was assessed by calculating coefficient of variation (CV%), with a cutoff of CV > 30% for exclusion unless a spot met predefined criteria for “Valid” intensity and shape.

Corrected peptide intensities were ranked from highest to lowest to identify dominant IgG-reactive epitopes for each pooled group. Additional visualization included heat-maps of spatial signal distribution from the upper left to lower right of the microarray to evaluate global uniformity and background noise.

### Data interpretation and statistical analysis

Signal intensities obtained from samples in each evaluated group are presented as a single value expressed in A.U., defined as the ratio between the fluorescence detected on microarrays incubated with serum samples and the corresponding background signal obtained from arrays exposed to the secondary antibody alone, as described above. All IgG–epitope interactions detected under these conditions were included in the analysis and are represented as a single quantitative value for each epitope within each donor group. Epitopes showing no detectable reactivity in any group were excluded from graphical visualization. **Supplementary Table S1** (Tab 2) lists exclusively the epitopes that exhibited measurable IgG binding and that are depicted in the Results section of this manuscript. Similarity clustering was performed using the IEDB Epitope Clustering Tool with an 80% identity threshold to indicate epitopes that may potentially be cross-recognized by IgG molecules. Statistical analyses were conducted using paired t-tests for comparisons involving matched samples, assuming an approximately Gaussian data distribution. All tests were two-tailed, and statistical significance was defined as p < 0.05. Data normality was evaluated by visual inspection of distribution plots and further assessed using the Shapiro–Wilk test.

## Results

### HTLV-1 patients produce differential patterns of viral epitope recognition

**Figure 1:**
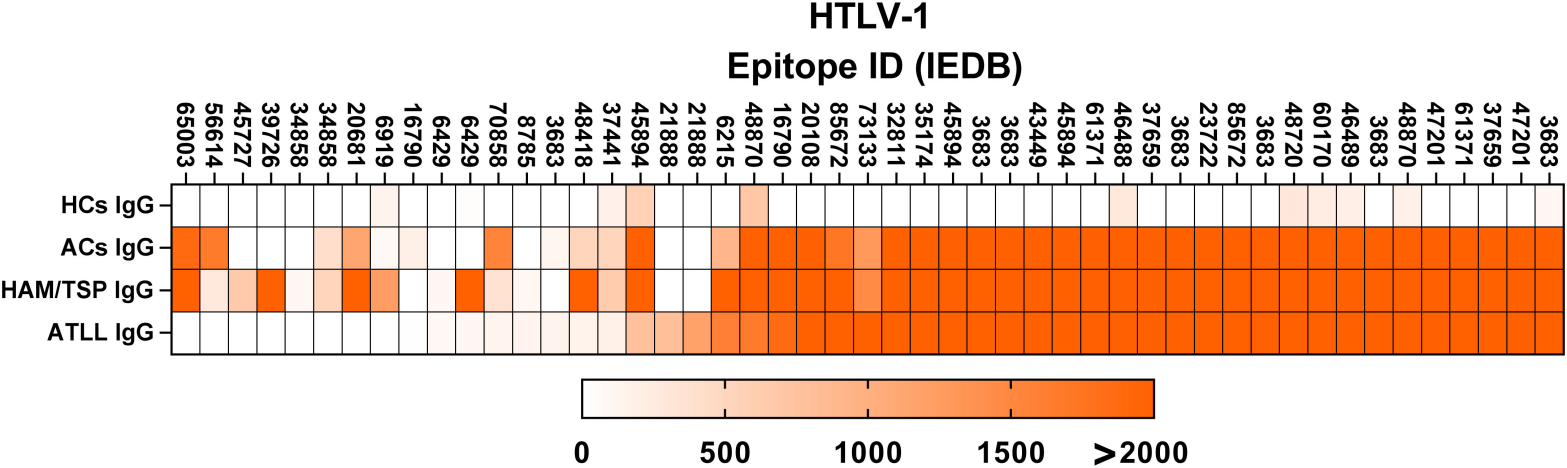
IgG recognition of HTLV-1–derived epitopes across clinical groups. This figure summarizes the results of Infectious Disease Epitope Microarray profiling performed using serum samples from four donor groups: healthy controls (HC IgG), asymptomatic carriers (AC IgG), individuals with HTLV-1–associated myelopathy/tropical spastic paraparesis (HAM/TSP IgG), and patients with adult T-cell leukemia/lymphoma (ATLL IgG). Heatmaps depict IgG reactivity across the complete panel of HTLV-1–derived linear epitopes. Epitopes are ordered from highest to lowest reactivity based on signal intensity observed in the ATLL IgG group. Signal intensities are expressed in arbitrary units (A.U.), calculated as the ratio between fluorescence detected following serum incubation and the corresponding background signal for each epitope. Color scaling ranges from white (0 A.U.) to intense orange (>2,000 A.U.). Epitopes are identified by their corresponding epitope identification numbers from the Immune Epitope Database (IEDB; iedb.org).

All serum samples were initially evaluated for IgG reactivity against 4,345 linear epitopes derived from viral, bacterial, parasitic, and fungal pathogens. To verify assay performance, IgG binding to 49 HTLV-1–specific epitopes was first assessed as an internal validation step. As shown in **Figure 1**, all HTLV-1–infected individuals (ACs, HAM/TSP, and ATLL) exhibited strong reactivity to 44 epitopes, whereas healthy controls (HCs) displayed only low-intensity binding restricted to 9 epitopes, resulting in a highly significant difference (p < 0.001) for all HTLV-1–infected groups compared with HCs.

When comparisons were performed among HTLV-1–infected groups, a statistically significant difference was observed between the AC and HAM/TSP groups (p < 0.001), whereas no significant difference was detected between the AC and ATLL groups. These findings indicate that both the pattern and intensity of HTLV-1 epitope recognition differ not only between infected and non-infected individuals but also among specific HTLV-1–associated clinical groups.

Although not intended for diagnostic use, these results confirm the expected recognition patterns, suggest the potential for group-level differentiation among infected individuals, and underscore the specificity and technical robustness of the epitope-profiling platform. Notably, distinct IgG reactivities against individual HTLV-1 epitopes were observed in clinically relevant groups: epitope 21888 was exclusively recognized by IgG from the ATLL group, whereas epitopes 39726 and 45727 were selectively recognized by IgG from the HAM/TSP group.

**Figure 2:**
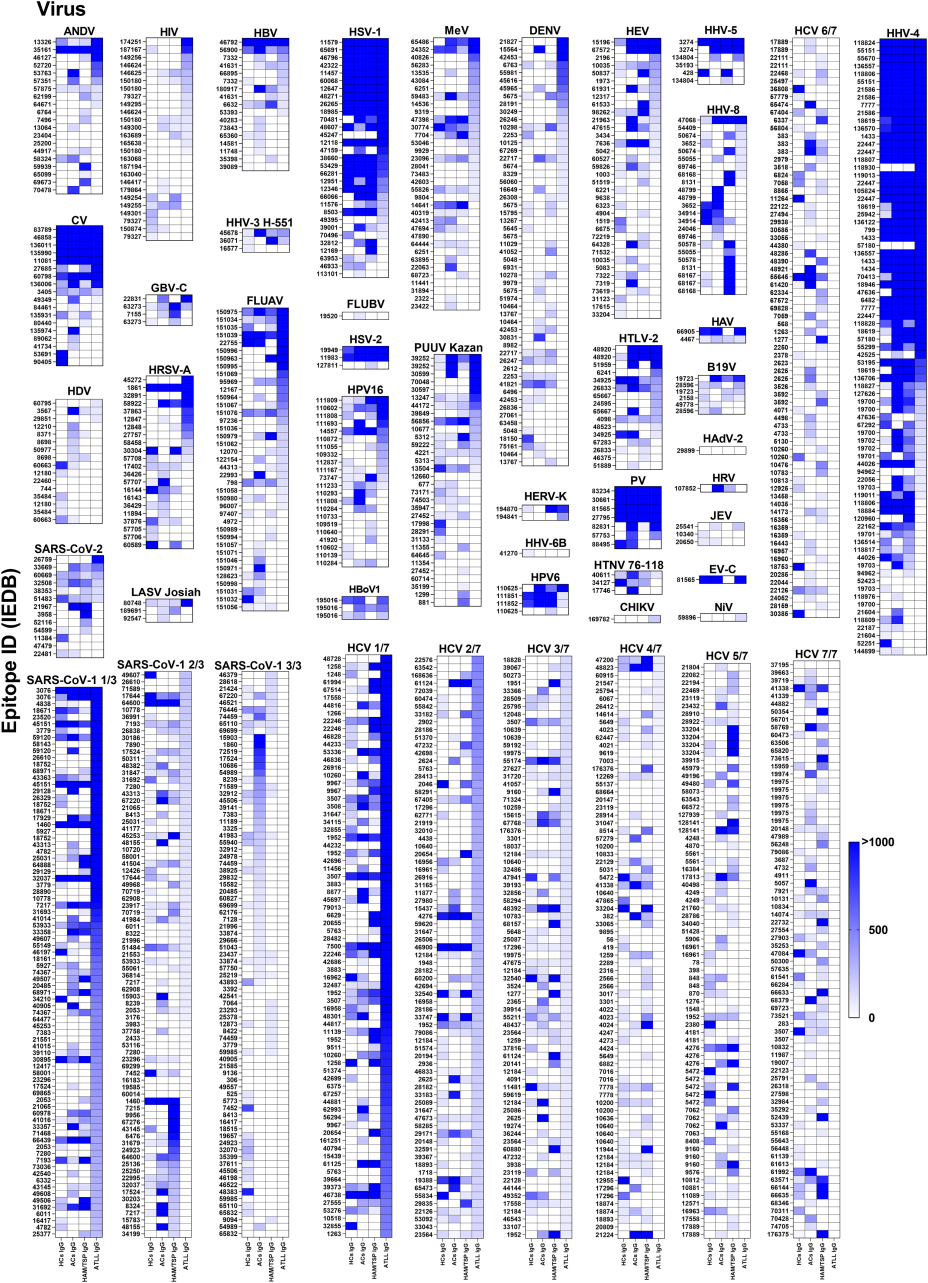
IgG Recognition of Viral Epitopes Across Distinct Donor Groups. Data were generated using the Infectious Disease Epitope Microarray to profile serum or purified IgG from four donor groups: healthy controls (HC IgG), asymptomatic carriers (AC IgG), individuals with HTLV-1–associated myelopathy/tropical spastic paraparesis (HAM/TSP IgG), and patients with adult T-cell leukemia/lymphoma (ATLL IgG). Heatmaps depict IgG reactivity across the complete panel of HTLV-1–derived linear epitopes. Epitopes are ordered from highest to lowest reactivity based on the signal intensity observed in the ATLL IgG group. Signal intensities are expressed in arbitrary units (A.U.), calculated as the ratio between fluorescence detected following serum incubation and the corresponding background signal for each epitope. Color scaling ranges from white (0 A.U.) to intense blue (>1,000 A.U.). Epitopes are identified by their corresponding epitope identification numbers from the Immune Epitope Database (IEDB; iedb.org). The pathogen of origin is indicated at the top of each individual heatmap; pathogens represented by a large number of epitopes were divided into multiple sequential panels (e.g., 1/3 to 3/3 or 1/7 to 7/7) for visualization purposes.

Building on this validation, we next examined IgG responses to epitopes from 52 additional viruses. Divergent recognition profiles emerged across all clinical groups, with measurable reactivity to at least one epitope from 39 viruses (**Figure 2**). The viruses with the highest number of recognized epitopes were SARS-CoV-1, HCV, and HHV-4. Although all groups recognized multiple epitopes from SARS-CoV-1 and HCV, the breadth and magnitude of IgG binding varied substantially between HTLV-1–infected groups and healthy controls. These differences reached statistical significance (FDR < 0.05) across all group comparisons. A comparable pattern was observed for influenza A virus (FLUAV), in which ATLL patients exhibited significantly distinct reactivity compared with healthy controls.

While several additional viral panels did not meet the statistical significance threshold, the heatmaps in **Figure 2** consistently demonstrate group-specific divergence in both epitope selection and signal intensity, reinforcing the notion of broad alterations in virus-specific IgG repertoires in HTLV-1 infection.

### HTLV-1 patients produce differential patterns of bacterial, parasitic, and fungal epitope recognition

**Figure 3:**
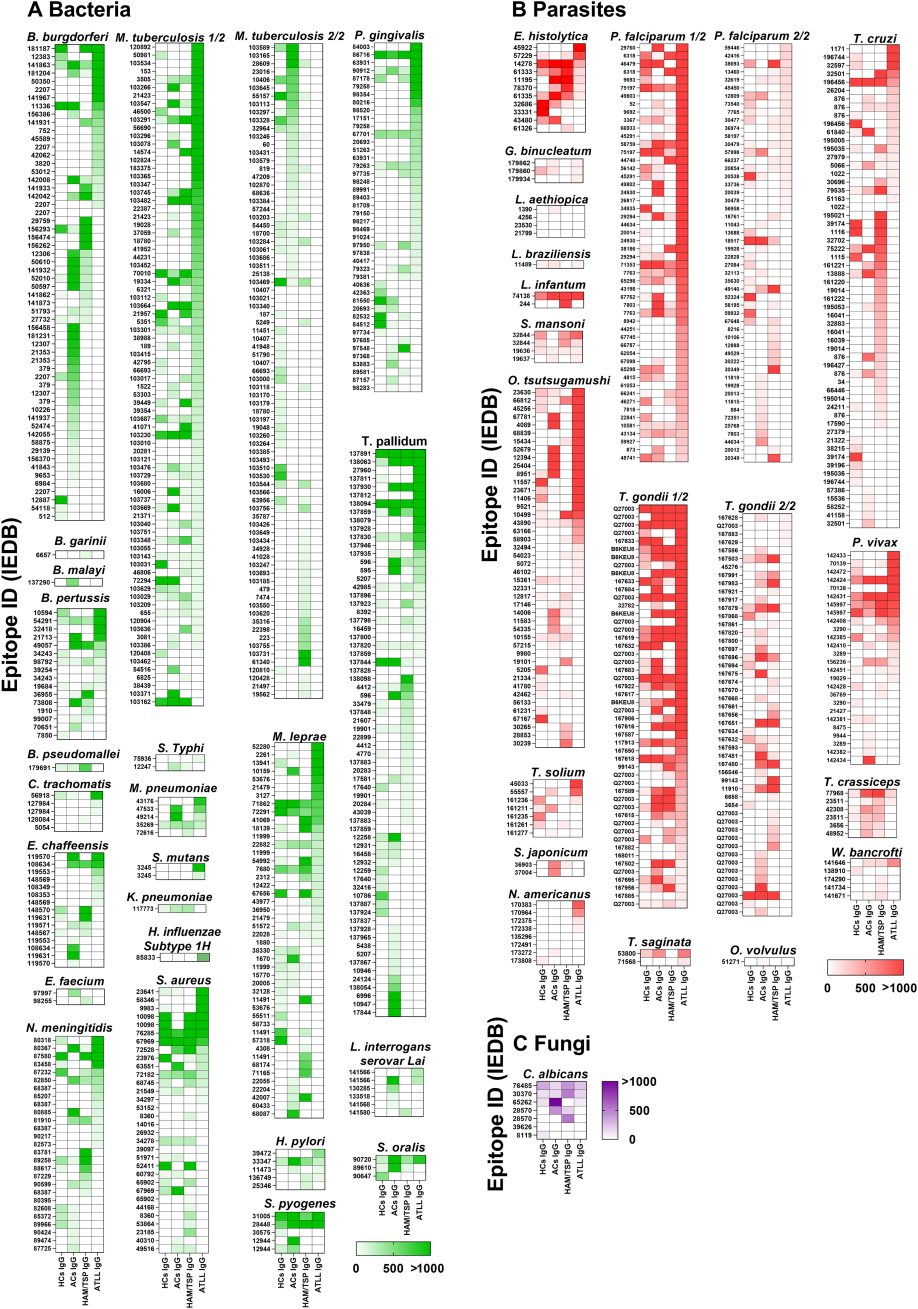
IgG Recognition of Bacterial, Parasitic, and Fungal Epitopes Across Distinct Donor Groups. Data were generated using the Infectious Disease Epitope Microarray to profile serum or purified IgG from four donor groups: healthy controls (HC IgG), asymptomatic carriers (AC IgG), individuals with HTLV-1–associated myelopathy/tropical spastic paraparesis (HAM/TSP IgG), and patients with adult T-cell leukemia/lymphoma (ATLL IgG). Heatmaps depict IgG reactivity across the complete panel of HTLV-1–derived linear epitopes. Epitopes are ordered from highest to lowest reactivity based on the signal intensity observed in the ATLL IgG group. Signal intensities are expressed in arbitrary units (A.U.), calculated as the ratio between fluorescence detected following serum incubation and the corresponding background signal for each epitope. Color scaling ranges from white (0 A.U.) to intense green for bacterial-origin epitopes **(A)**, intense red for parasite-origin epitopes **(B)**, and intense purple for fungal-origin epitopes **(C)**, with values exceeding 1,000 A.U. Epitopes are identified by their corresponding epitope identification numbers from the Immune Epitope Database (IEDB; iedb.org). The pathogen of origin is indicated at the top of each individual heatmap; pathogens represented by a large number of epitopes were divided into multiple sequential panels (e.g., 1/2 to 2/2) for visualization purposes.

To determine whether these group-specific differences extended beyond viral antigens, we next analyzed IgG binding to bacterial epitopes (**Figure 3A**). Distinct recognition patterns were observed for 23 bacterial species. Among these, *Mycobacterium tuberculosis* exhibited the highest number and intensity of recognized epitopes, with statistically significant differences between ATLL patients and healthy controls. A similar pattern was seen for Borrelia burgdorferi, where all HTLV-1–infected groups demonstrated significantly stronger IgG reactivity than healthy controls. Although other bacterial panels did not reach statistical significance, the overall response profiles clearly indicate systematic divergence in the breadth and magnitude of IgG recognition across groups.

We then extended this analysis to parasitic epitopes (**Figure 3B**), where similarly heterogeneous and group-specific patterns emerged. Divergent recognition profiles were identified for 16 parasitic species, with *Plasmodium falciparum* and *Toxoplasma gondii* eliciting the strongest IgG responses. Notably, *T. gondii* recognition was significantly higher across all HTLV-1–infected groups compared with healthy controls, whereas statistically significant differences for *P. falciparum* and *P. vivax* were restricted to comparisons between ATLL patients and healthy controls. As observed for viral and bacterial epitopes, the remaining parasitic epitope sets—although not statistically significant—still revealed consistent qualitative differences in the targets and intensity of IgG binding among groups.

Finally, when examining fungal epitopes, divergent IgG recognition was detected only for *Candida albicans*, although these differences did not reach statistical significance. Even so, the pattern mirrored the broader trend of subtle but consistent shifts in epitope recognition across the HTLV-1 clinical spectrum.

### Epitope similarity analysis for cross-reactivity prediction

To determine whether the observed IgG repertoires might reflect simple sequence-based cross-reactivity, we performed similarity clustering of all recognized epitopes using an 80% identity threshold. This approach allowed us to evaluate whether IgG reactivity could be attributed to shared structural features rather than distinct antigenic exposures.

**Figure 4:**
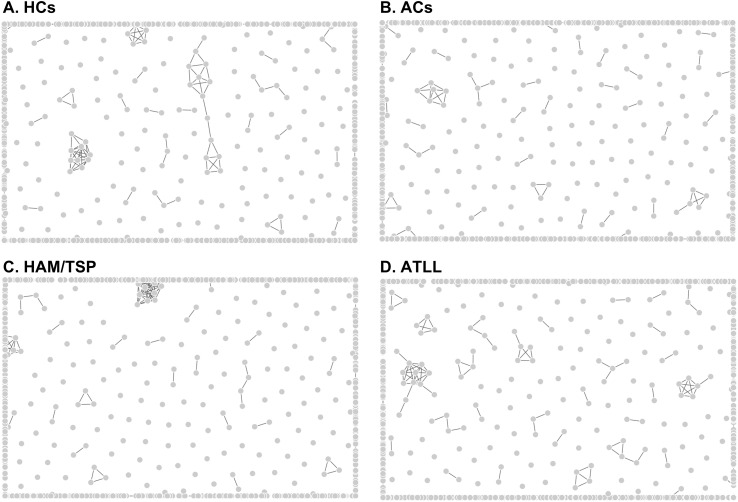
Epitope similarity clustering. All IgG-recognized epitope sequences from each donor group were analyzed for sequence similarity using the IEDB Epitope Clustering Tool, applying an 80% identity threshold with no size restrictions. Each dot represents a single epitope, and edges indicate sequence similarity between epitope pairs. Sparse or absent similarity results in peripheral dispersion of points, whereas densely interconnected epitopes form central clusters, reflecting higher degrees of shared sequence identity. Panels depict clustering patterns for epitopes recognized by IgG from healthy controls [HCs, **(A)**], asymptomatic carriers [ACs, **(B)**], individuals with HTLV-1–associated myelopathy/tropical spastic paraparesis [HAM/TSP, **(C)**], and patients with adult T-cell leukemia/lymphoma [ATLL, **(D)**].

In healthy controls, 1,041 unique epitopes formed 750 similarity clusters (**Figure 4A**). ACs recognized 1,183 epitopes, which clustered into 841 groups (**Figure 4B**). HAM/TSP patients recognized 1,146 epitopes forming 810 clusters (**Figure 4C**), whereas ATLL patients recognized the largest repertoire—1,286 epitopes—forming 895 clusters (**Figure 4D**). Comprehensive peptide sequences and cluster assignments are provided in **Supplementary Tables S2–S5**.

Across all groups, the large number of clusters relative to the number of epitopes indicates low sequence redundancy and therefore a low likelihood that the observed IgG profiles result from simple cross-reactivity. Instead, these findings suggest that HTLV-1 infection—and its associated clinical manifestations—may shape distinct and biologically meaningful IgG epitope repertoires across a wide range of pathogens.

## Discussion

In this study, we employed a high-density infectious disease epitope microarray to characterize IgG epitope-recognition profiles in individuals with distinct clinical outcomes of HTLV-1 infection. This approach enabled a comprehensive assessment of humoral immune recognition across a broad spectrum of pathogen-derived epitopes while also allowing validation of HTLV-1–specific responses within the same analytical framework.

Before undertaking the broad evaluation of pathogen recognition, our initial analysis of IgG reactivity against HTLV-1–derived epitopes provided clear evidence of the robustness and reliability of the experimental approach. This was demonstrated by the unequivocal discrimination between HTLV-1–infected and non-infected individuals. Moreover, distinct epitope-specific IgG responses were observed among clinically relevant HTLV-1 groups: epitope 21888 was exclusively recognized by IgG from individuals with ATLL, whereas epitopes 39726 and 45727 were selectively recognized by IgG from individuals with HTLV-1–associated HAM/TSP.

According to annotations from the Immune Epitope Database (IEDB) and UniProt databases, epitope 21888 is derived from the HTLV-1 Gag–Pro–Pol polyprotein, which is generated through a translational readthrough event during viral RNA translation. This process produces a single elongated polyprotein that integrates the products of the gag (group-specific antigen), pro (protease), and pol (polymerase) genes. The Gag–Pro–Pol polyprotein plays a critical structural role in the assembly of new viral particles while simultaneously harboring the enzymatic functions required for viral maturation and subsequent infection of new target cells.

In contrast, epitopes 39726 and 45727 are derived from an HTLV-1 envelope glycoprotein that is essential for viral entry, cell-to-cell transmission, and immune evasion. This envelope protein mediates binding to host cells, triggers membrane fusion, promotes syncytium formation, and represents a highly conserved viral component as well as a primary target for neutralizing antibody responses. The selective recognition of these envelope-derived epitopes by IgG from HAM/TSP individuals suggests disease-associated differences in humoral targeting of viral antigens.

Although the present study was not designed to validate individual viral proteins or epitopes as diagnostic markers for HTLV-1–associated clinical conditions, these findings nonetheless highlight the potential biological relevance of disease-specific IgG recognition patterns. Collectively, these observations suggest that epitope-level profiling may capture clinically meaningful differences in humoral immune responses among HTLV-1–infected individuals and may inform future studies aimed at dissecting the relationship between viral antigen recognition, immune dysregulation, and disease pathogenesis.

By quantifying IgG responses to more than 4,300 pathogen-derived linear epitopes, we identified extensive alterations in the breadth, diversity, and intensity of humoral recognition associated with ACs, HAM/TSP, and ATLL. These findings indicate that HTLV-1 infection does not merely elicit virus-specific immune responses but reconfigures the humoral landscape at a systems level.

The immune system of HTLV-1–infected individuals is characterized by persistent activation, even among clinically asymptomatic carriers. The viral regulatory proteins Tax and HBZ chronically stimulate NF-κB, STAT1, AP-1, and IRF signaling, leading to sustained transcription of proinflammatory mediators and abnormal T-cell proliferation (35, 36).

Against this backdrop, the pronounced divergence of IgG repertoires across clinical groups observed in our study is unsurprising. Yet, the extent of divergence—including responses to pathogens unrelated to HTLV-1—reveals a level of systemic immunological remodeling that exceeds previous descriptions.

Our findings further indicate that each clinical phenotype displays a distinctive antibody-recognition signature. HAM/TSP is marked by high proviral load, heightened Th1 activation, and elevated IFN-γ production (37), conditions known to promote broad antigen recognition, enhanced B-cell maturation, and robust inflammatory antibody responses. Consistent with this profile, HAM/TSP participants exhibited greater intensity and diversity of IgG recognition across viral, bacterial, and parasitic epitopes. Conversely, ATLL reflects a profoundly different immunological state, dominated by malignant transformation, transcriptional reprogramming, immunosuppression, and defective antigen presentation (38). Although ATLL patients recognized the largest number of epitopes, their pattern of reactivity was distinct, suggesting dysregulated or atypical B-cell activation. This observation mirrors transcriptomic evidence demonstrating that ATLL cells disrupt immune communication networks and secrete immunosuppressive cytokines that modulate B-cell development and antibody output (36).

We additionally observed heightened IgG responses to pathogens frequently associated with co-infections in HTLV-1. Elevated reactivity against *Mycobacterium tuberculosis, Strongyloides stercoralis, Toxoplasma gondii*, and *Plasmodium* species aligns with clinical data showing that HTLV-1 infection predisposes individuals to severe strongyloidiasis, tuberculosis, and toxoplasmosis (39). These amplified antibody responses may reflect repeated exposure, impaired pathogen control, or chronic antigenic stimulation; however, in the absence of corresponding clinical or microbiological testing, these possibilities cannot be definitively established. The IFN-γ–driven environment characteristic of HTLV-1 infection facilitates intracellular pathogen persistence, likely contributing to sustained IgG production.

For pathogens such as SARS-CoV-related viruses, HCV, EBV (HHV-4), and influenza, the group-specific IgG differences may partly reflect heterogeneous exposure histories. However, the magnitude and consistency of divergence are more consistent with altered B-cell selection, activation thresholds, or memory-cell maintenance driven by HTLV-1–mediated immune dysregulation.

Beyond known co-infections, our results provide compelling evidence for heterologous immunity—whereby immune responses to one pathogen shape subsequent responses to unrelated antigens (40). Heterologous immunity can have protective or pathogenic consequences depending on context (41). In HTLV-1 infection, chronic antigenic stimulation, dysregulated costimulatory signaling, and persistent inflammation may collectively prime B-cell memory pools, generating widespread alterations in epitope recognition—even against pathogens not recently encountered.

Analogous patterns have been described in other chronic viral infections. HIV, for example, induces hypergammaglobulinemia, clonal B-cell expansion, and diversification of antibody repertoires (42). Although HTLV-1 operates through distinct mechanisms, our findings suggest that it similarly contributes to increased immune turnover and activation of noncanonical B-cell pathways.

A particularly notable finding is that IgG responses within each group comprised hundreds of clusters with minimal sequence similarity. This observation argues against simple linear cross-reactivity and instead points toward altered B-cell selection dynamics, impaired tolerance, clonal expansions, and systemic perturbations of antibody homeostasis.

This broader perspective aligns with idiotypic network theory, first proposed by Jerne (43) and later expanded into modern models describing antibody–antibody regulatory circuits (44). Recent large-scale sequencing studies further demonstrate that healthy individuals maintain highly structured and self-regulated antibody repertoires (45). HTLV-1, by altering lymphocyte homeostasis and inducing chronic immune activation, may destabilize these regulatory networks.

This interpretation also resonates with the “hooks without bait” hypothesis (13), which proposes that pathological states associated with infections such as HIV (24) and COVID-19 (16, 32, 33) can generate IgG idiotype repertoires capable of interacting with self-proteins expressed on immune cells or in soluble form, thereby modulating immune responses. Notably, IgG-mediated immunomodulatory effects have also been documented in non-infectious conditions, including atopic dermatitis (19–21, 46–49), allergic diseases such as dust-mite allergy (15, 18, 22, 23, 25, 50–52), and in HTLV-1 infection itself—where distinct clinical phenotypes have been shown to associate with divergent autoreactive IgG repertoires possessing immunomodulatory potential (30). Our observations strongly parallel these findings, suggesting that shifts in idiotype networks may contribute to the clinical heterogeneity of HTLV-1 disease.

Collectively, our results demonstrate that HTLV-1 infection drives widespread remodeling of IgG epitope recognition that cannot be attributed solely to antigen exposure. Instead, these patterns likely arise from the intersection of chronic infection, persistent inflammation, B-cell dysregulation, and impaired idiotypic network regulation.

These findings carry several important implications. First, distinct IgG repertoires across ACs, HAM/TSP, and ATLL patients may serve as biomarkers capable of stratifying clinical phenotypes. Second, enhanced IgG recognition of specific pathogens corresponds to known clinical vulnerabilities and may support improved co-infection surveillance strategies. Third, the low sequence similarity among epitopes highlights the need to investigate structural, conformational, and post-translational determinants of antibody binding in HTLV-1. Fourth, therapeutic strategies aimed at reducing chronic inflammation or restoring B-cell regulatory circuits may prove beneficial. Finally, future studies integrating epitope profiling with deep BCR sequencing, single-cell immunophenotyping, and longitudinal sampling will be essential to determine how humoral signatures evolve over time and whether they can predict progression to HAM/TSP or ATLL.

This study was designed to provide a comprehensive descriptive analysis of IgG epitope-recognition patterns across distinct clinical outcomes of HTLV-1 infection. While the cross-sectional nature of the cohort limits causal inference and longitudinal interpretation, the standardized experimental conditions and consistent group-level patterns support the robustness of the observed findings. Future studies incorporating longitudinal sampling and functional assays may further clarify the mechanistic implications of these humoral signatures.

## Data Availability

The raw data supporting the conclusions of this article will be made available by the authors, without undue reservation.
