# Supplementary material for: Clinical outcomes-dependent IgG epitope profiling in HTLV-1 reveals differential recognition of pathogen-derived antigens
Source: Front Immunol. 2026 Feb 25;17:1755133. doi: 10.3389/fimmu.2026.1755133 (PMC12975881; doi:10.3389/fimmu.2026.1755133)
Supplement: Supplementary file 2 [file Table2.docx]

| **Cluster.Sub-Cluster Number** | **Peptide Number** | **Alignment** | **Position** | **Description** | **Peptide** |
| --- | --- | --- | --- | --- | --- |
| 1.1 | Consensus | GKPAIIPDREVLYREFDEMEE | - | - | - |
| 1.1 | 1 | GKPAIIPDRE----------- | 1 | seq147 | GKPAIIPDRE |
| 1.1 | 2 | GKPAIIPDR------------ | 1 | seq144 | GKPAIIPDR |
| 1.1 | 3 | -KPAIIPDREVLYREF----- | 2 | seq116 | KPAIIPDREVLYREF |
| 1.1 | 4 | -KPAIIPDREVLYQEF----- | 2 | seq146 | KPAIIPDREVLYQEF |
| 1.1 | 5 | -KPAIIPDRE----------- | 2 | seq197 | KPAIIPDRE |
| 1.1 | 6 | --PAIIPDREV---------- | 3 | seq84 | PAIIPDREV |
| 1.1 | 7 | ---AIIPDREVL--------- | 4 | seq292 | AIIPDREVL |
| 1.1 | 8 | ----IIPDREVLYREFDEM-- | 5 | seq121 | IIPDREVLYREFDEM |
| 1.1 | 9 | ----VIPDREVLYR------- | 5 | seq604 | VIPDREVLYR |
| 1.1 | 10 | ----IIPDREVLY-------- | 5 | seq117 | IIPDREVLY |
| 1.1 | 11 | -----VPDREVLYREFDEM-- | 6 | seq448 | VPDREVLYREFDEM |
| 1.1 | 12 | -----IPDREVLYREFDEM-- | 6 | seq122 | IPDREVLYREFDEM |
| 1.1 | 13 | ------PDREVLYRE------ | 7 | seq87 | PDREVLYRE |
| 1.1 | 14 | -------DREVLYREF----- | 8 | seq642 | DREVLYREF |
| 1.1 | 15 | --------REVLYQEFDEMEE | 9 | seq451 | REVLYQEFDEMEE |
| 1.1 | 16 | --------REVLYREFDEME- | 9 | seq386 | REVLYREFDEME |
| 1.1 | 17 | --------REVLYREFD---- | 9 | seq348 | REVLYREFD |
| 1.1 | 18 | ---------EVLYREFDE--- | 10 | seq350 | EVLYREFDE |
| 1.1 | 19 | ----------VLYREFDEM-- | 11 | seq336 | VLYREFDEM |
| 1.1 | 20 | ----------VLYREFDE--- | 11 | seq371 | VLYREFDE |
| 1.1 | 21 | -----------LYREFD---- | 12 | seq631 | LYREFD |
| 2.1 | Consensus | SLYPGHXSGHRMAWDMMMNWSXTGQRMAWDMMMNWS | - | - | - |
| 2.1 | 1 | SLYPGHVSGHRMAWD--------------------- | 1 | seq881 | SLYPGHVSGHRMAWD |
| 2.1 | 2 | --YPGHVSGHRMAWDMM------------------- | 3 | seq1114 | YPGHVSGHRMAWDMM |
| 2.1 | 3 | ---PGHITGHRMAWDMMM------------------ | 4 | seq952 | PGHITGHRMAWDMMM |
| 2.1 | 4 | ---PGHVSGHRMAWDMMM------------------ | 4 | seq1049 | PGHVSGHRMAWDMMM |
| 2.1 | 5 | ------ITGHRMAWDMMLNWS--------------- | 7 | seq965 | ITGHRMAWDMMLNWS |
| 2.1 | 6 | ------ITGHRMAWDMMMNWS--------------- | 7 | seq897 | ITGHRMAWDMMMNWS |
| 2.1 | 7 | ------LSGHRMAWDMMMNWS--------------- | 7 | seq962 | LSGHRMAWDMMMNWS |
| 2.1 | 8 | ------ISGHRMAWDMMMNWS--------------- | 7 | seq1093 | ISGHRMAWDMMMNWS |
| 2.1 | 9 | -------SGHRMAWDMMMNWSP-------------- | 8 | seq728 | SGHRMAWDMMMNWSP |
| 2.1 | 10 | -------SGHRMAWDMMMNW---------------- | 8 | seq771 | SGHRMAWDMMMNW |
| 2.1 | 11 | -------SGHRMAWDM-------------------- | 8 | seq853 | SGHRMAWDM |
| 2.1 | 12 | --------GHRMAWDMM------------------- | 9 | seq993 | GHRMAWDMM |
| 2.1 | 13 | --------GHRMAWDM-------------------- | 9 | seq1078 | GHRMAWDM |
| 2.1 | 14 | ---------HRMAWDMMM------------------ | 10 | seq1042 | HRMAWDMMM |
| 2.1 | 15 | ---------HRMAWDMM------------------- | 10 | seq954 | HRMAWDMM |
| 2.1 | 16 | ----------RMAWDMMM------------------ | 11 | seq1111 | RMAWDMMM |
| 2.1 | 17 | ---------------------ITGQRMAWDMMMNWS | 22 | seq922 | ITGQRMAWDMMMNWS |
| 3.1 | Consensus | GGPDGEPDVPPGAIEQGPADDPGEGPSTGPRGQGDGGRRK | - | - | - |
| 3.1 | 1 | GGPDGEPDVPPGAIEQG----------------------- | 1 | seq60 | GGPDGEPDVPPGAIEQG |
| 3.1 | 2 | GGPDGEPDVPPGAIE------------------------- | 1 | seq40 | GGPDGEPDVPPGAIE |
| 3.1 | 3 | ---DGEPDVPPGAIEQGPAD-------------------- | 4 | seq45 | DGEPDVPPGAIEQGPAD |
| 3.1 | 4 | ---DGEPDVPPGAIEQGP---------------------- | 4 | seq49 | DGEPDVPPGAIEQGP |
| 3.1 | 5 | ----GEPDVPPGAIEQGPADD------------------- | 5 | seq61 | GEPDVPPGAIEQGPADD |
| 3.1 | 6 | -------DVPPGAIEQGPADDPGE---------------- | 8 | seq64 | DVPPGAIEQGPADDPGE |
| 3.1 | 7 | ----------PGAIEQGPADDPGEGPS------------- | 11 | seq90 | PGAIEQGPADDPGEGPS |
| 3.1 | 8 | -------------IEQGPADDPGEGPSTGP---------- | 14 | seq109 | IEQGPADDPGEGPSTGP |
| 3.1 | 9 | ---------------QGPADDPGEGPST------------ | 16 | seq118 | QGPADDPGEGPST |
| 3.1 | 10 | ----------------GPADDPGEGPSTG----------- | 17 | seq101 | GPADDPGEGPSTG |
| 3.1 | 11 | -----------------PADDPGEGPSTGPRGQG------ | 18 | seq85 | PADDPGEGPSTGPRGQG |
| 3.1 | 12 | -------------------DDPGEGPSTGPR--------- | 20 | seq76 | DDPGEGPSTGPR |
| 3.1 | 13 | --------------------DPGEGPSTGPRGQGDGG--- | 21 | seq97 | DPGEGPSTGPRGQGDGG |
| 3.1 | 14 | -----------------------EGPSTGPRGQGDGGRRK | 24 | seq291 | EGPSTGPRGQGDGGRRK |
| 4.1 | Consensus | APPLLPHSNLDHILEPSIPWKSK | - | - | - |
| 4.1 | 1 | APPLLPHSNLDHI---------- | 1 | seq17 | APPLLPHSNLDHI |
| 4.1 | 2 | ---LLPHSNLDHILEPSI----- | 4 | seq23 | LLPHSNLDHILEPSI |
| 4.1 | 3 | ---LLPHSNLD------------ | 4 | seq56 | LLPHSNLD |
| 4.1 | 4 | ----LPHSLNDHILEPSIP---- | 5 | seq79 | LPHSLNDHILEPSIP |
| 4.1 | 5 | ----LPHSNL------------- | 5 | seq39 | LPHSNL |
| 4.1 | 6 | -----PHSNLDHI---------- | 6 | seq52 | PHSNLDHI |
| 4.1 | 7 | -----PHSNLDH----------- | 6 | seq63 | PHSNLDH |
| 4.1 | 8 | ------HSNLDHILEPSIPWK-- | 7 | seq46 | HSNLDHILEPSIPWK |
| 4.1 | 9 | ------HSNLDHI---------- | 7 | seq59 | HSNLDHI |
| 4.1 | 10 | --------LNDHILEPSIPWKSK | 9 | seq65 | LNDHILEPSIPWKSK |
| 4.1 | 11 | ---------LDHILEPSIPWKSK | 10 | seq72 | LDHILEPSIPWKSK |
| 4.2 | Singleton | PPTAPPLLPHSNL | - | seq99 | PPTAPPLLPHSNL |
| 5.1 | Consensus | SGSPPRRPPPGRRPFFHPVGEADYFEYHQE | - | - | - |
| 5.1 | 1 | SGSPPRRPPPGRRPFFH------------- | 1 | seq210 | SGSPPRRPPPGRRPFFH |
| 5.1 | 2 | --SPPRRPPPGRRPFFHPV----------- | 3 | seq139 | SPPRRPPPGRRPFFHPV |
| 5.1 | 3 | ---PPRRPPPGRRPFFHPVG---------- | 4 | seq168 | PPRRPPPGRRPFFHPVG |
| 5.1 | 4 | ---PPRRPPPGRRPFF-------------- | 4 | seq103 | PPRRPPPGRRPFF |
| 5.1 | 5 | -----RRPPPGRRPFFHPVGEA-------- | 6 | seq196 | RRPPPGRRPFFHPVGEA |
| 5.1 | 6 | -------PPPGRRPFFHPVGE--------- | 8 | seq188 | PPPGRRPFFHPVGE |
| 5.1 | 7 | --------PPGRRPFFHPVGEADYF----- | 9 | seq111 | PPGRRPFFHPVGEADYF |
| 5.1 | 8 | ----------GRRPFFHPVGEADYFEY--- | 11 | seq7 | GRRPFFHPVGEADYFEY |
| 5.1 | 9 | ----------GRRPFFHPVGE--------- | 11 | seq234 | GRRPFFHPVGE |
| 5.1 | 10 | -----------RRPFFHPVGEADYFEYH-- | 12 | seq22 | RRPFFHPVGEADYFEYH |
| 5.1 | 11 | -------------PFFHPVGEADYFEYHQE | 14 | seq26 | PFFHPVGEADYFEYHQE |
| 6.1 | Consensus | GDKPSXFGQAAAGDKPSLFGQAAAGDKPPP | - | - | - |
| 6.1 | 1 | GDKPSLFGQAAAGDK--------------- | 1 | seq592 | GDKPSLFGQAAAGDK |
| 6.1 | 2 | ---PSPFGQAAAGDK--------------- | 4 | seq545 | PSPFGQAAAGDK |
| 6.1 | 3 | ----SLFGQAAAGDKPSLF----------- | 5 | seq473 | SLFGQAAAGDKPSLF |
| 6.1 | 4 | -----PFGQAAAGDKPS------------- | 6 | seq331 | PFGQAAAGDKPS |
| 6.1 | 5 | ------FGQAAAGDKLSL------------ | 7 | seq469 | FGQAAAGDKLSL |
| 6.1 | 6 | ------FGQAAAGDKPAP------------ | 7 | seq368 | FGQAAAGDKPAP |
| 6.1 | 7 | ------FGQAAAGDKPSL------------ | 7 | seq577 | FGQAAAGDKPSL |
| 6.1 | 8 | ------FGQAAAGDK--------------- | 7 | seq587 | FGQAAAGDK |
| 6.1 | 9 | --------QAAAGDKPS------------- | 9 | seq538 | QAAAGDKPS |
| 6.1 | 10 | ---------AAAGDK--------------- | 10 | seq706 | AAAGDK |
| 6.1 | 11 | ------------------FGQAAAGDKPPP | 19 | seq493 | FGQAAAGDKPPP |
| 7.1 | Consensus | RXRGRGRGGGGGRPGAPGGS | - | - | - |
| 7.1 | 1 | RGRGRGRGRGGGRPGAP--- | 1 | seq412 | RGRGRGRGRGGGRPGAP |
| 7.1 | 2 | -RGRGRGRGGGGRPGAP--- | 2 | seq340 | RGRGRGRGGGGRPGAP |
| 7.1 | 3 | --RGRGGRGGGGRPGAP--- | 3 | seq391 | RGRGGRGGGGRPGAP |
| 7.1 | 4 | --RGRGRGGGGGRPGAP--- | 3 | seq395 | RGRGRGGGGGRPGAP |
| 7.1 | 5 | ---GRGRGRGGGRPGAPGGS | 4 | seq491 | GRGRGRGGGRPGAPGGS |
| 7.1 | 6 | ------RGRGGGRPGAPG-- | 7 | seq504 | RGRGGGRPGAPG |
| 7.1 | 7 | ------GGGGGGGPGAP--- | 7 | seq561 | GGGGGGGPGAP |
| 7.1 | 8 | -------RGGGGRPGAPG-- | 8 | seq486 | RGGGGRPGAPG |
| 7.1 | 9 | --------GGGGRPGAPG-- | 9 | seq558 | GGGGRPGAPG |
| 7.1 | 10 | ---------GGGGPGAPG-- | 10 | seq722 | GGGGPGAPG |
| 8.1 | Consensus | KTKRNTNRRPQDVKFPGGGQI | - | - | - |
| 8.1 | 1 | KTKRNTNRRPQDVKF------ | 1 | seq36 | KTKRNTNRRPQDVKF |
| 8.1 | 2 | ---RNTNRRPQDVKF------ | 4 | seq19 | RNTNRRPQDVKF |
| 8.1 | 3 | -----TNRRPQDVKFPGGGQ- | 6 | seq155 | TNRRPQDVKFPGGGQ |
| 8.1 | 4 | -------RRPQDVKFPG---- | 8 | seq142 | RRPQDVKFPG |
| 8.1 | 5 | -------RRPQDVKF------ | 8 | seq12 | RRPQDVKF |
| 8.1 | 6 | --------RPQDVKFP----- | 9 | seq50 | RPQDVKFP |
| 8.1 | 7 | ---------PQDVKFPGGGQI | 10 | seq169 | PQDVKFPGGGQI |
| 8.1 | 8 | ---------PQDVKFPGGG-- | 10 | seq195 | PQDVKFPGGG |
| 8.1 | 9 | -----------DVKFPGGGQI | 12 | seq714 | DVKFPGGGQI |
| 9.1 | Consensus | GPTHSKEIPALTAVETGATNPX | - | - | - |
| 9.1 | 1 | GPTHSKEIPALTAVE------- | 1 | seq58 | GPTHSKEIPALTAVE |
| 9.1 | 2 | ---HSKEIPALTAVETGA---- | 4 | seq27 | HSKEIPALTAVETGA |
| 9.1 | 3 | -----KEVPALTAVETGAT--- | 6 | seq34 | KEVPALTAVETGAT |
| 9.1 | 4 | ------EIPALTAVETGATNP- | 7 | seq41 | EIPALTAVETGATNP |
| 9.1 | 5 | -------IPALTAAETGHTSQV | 8 | seq24 | IPALTAAETGHTSQV |
| 9.1 | 6 | -------VPALTAVETGHT--- | 8 | seq37 | VPALTAVETGHT |
| 9.1 | 7 | --------PALTAVETGATNPL | 9 | seq43 | PALTAVETGATNPL |
| 9.1 | 8 | --------PALTAAETG----- | 9 | seq31 | PALTAAETG |
| 9.2 | Singleton | LTAVETGHTSQV | - | seq163 | LTAVETGHTSQV |
| 10.1 | Consensus | APDKEILYEAFDEM | - | - | - |
| 10.1 | 1 | APDKEVLYEAFDEM | 1 | seq459 | APDKEVLYEAFDEM |
| 10.1 | 2 | APDKEILYEAFDEM | 1 | seq484 | APDKEILYEAFDEM |
| 10.1 | 3 | TPDKEILYE----- | 1 | seq688 | TPDKEILYE |
| 10.1 | 4 | --DKEILYEAF--- | 3 | seq725 | DKEILYEAF |
| 10.1 | 5 | ---KEILYEAFD-- | 4 | seq535 | KEILYEAFD |
| 10.1 | 6 | ----EILYEAFDE- | 5 | seq343 | EILYEAFDE |
| 10.1 | 7 | -----ILYEAFDEM | 6 | seq392 | ILYEAFDEM |
| 10.2 | Singleton | VTPDKEILY | - | seq1083 | VTPDKEILY |
| 11.1 | Consensus | SSIVYEAADMIMHTP | - | - | - |
| 11.1 | 1 | SSIVYEAADAILHT- | 1 | seq153 | SSIVYEAADAILHT |
| 11.1 | 2 | SSIVYEAADMIMHT- | 1 | seq86 | SSIVYEAADMIMHT |
| 11.1 | 3 | ---VYEAADMIM--- | 4 | seq140 | VYEAADMIM |
| 11.1 | 4 | ----YEAADMIMHTP | 5 | seq172 | YEAADMIMHTP |
| 11.1 | 5 | ----YEAADAILHTP | 5 | seq413 | YEAADAILHTP |
| 11.2 | Singleton | SSIVYEADDVILHT | - | seq74 | SSIVYEADDVILHT |
| 12.1 | Consensus | GGAGAGGGAGGAGAGGGAGGAGX | - | - | - |
| 12.1 | 1 | GGAGAGGGAGGAGAGGG------ | 1 | seq83 | GGAGAGGGAGGAGAGGG |
| 12.1 | 2 | --AGAGGGAGGAGAGGGAG---- | 3 | seq57 | AGAGGGAGGAGAGGGAG |
| 12.1 | 3 | ---GAGGGAGGAGAGGGAGG--- | 4 | seq98 | GAGGGAGGAGAGGGAGG |
| 12.1 | 4 | -----GGGAGGAGAGGGAGGAG- | 6 | seq93 | GGGAGGAGAGGGAGGAG |
| 12.1 | 5 | ------GGAGGAGAGGGAGGAGG | 7 | seq89 | GGAGGAGAGGGAGGAGG |
| 12.1 | 6 | ------GGAGGAGAGGGAGGAGA | 7 | seq66 | GGAGGAGAGGGAGGAGA |
| 13.1 | Consensus | QAAPYIEQAQVI | - | - | - |
| 13.1 | 1 | QAAPYIEQA--- | 1 | seq492 | QAAPYIEQA |
| 13.1 | 2 | -AAPYIEQAQ-- | 2 | seq639 | AAPYIEQAQ |
| 13.1 | 3 | --APYIEQAQV- | 3 | seq685 | APYIEQAQV |
| 13.1 | 4 | ---PYIEQAQVI | 4 | seq568 | PYIEQAQVI |
| 13.2 | Consensus | CSQAAPYIEQ | - | - | - |
| 13.2 | 1 | CSQAAPYIE- | 1 | seq829 | CSQAAPYIE |
| 13.2 | 2 | -SQAAPYIEQ | 2 | seq347 | SQAAPYIEQ |
| 14.1 | Consensus | VEEQEQEQEEQELEEVEEQEQE | - | - | - |
| 14.1 | 1 | VEEQEQEQEEQELEE------- | 1 | seq287 | VEEQEQEQEEQELEE |
| 14.1 | 2 | VEEQEQEQEEQEEQE------- | 1 | seq233 | VEEQEQEQEEQEEQE |
| 14.1 | 3 | --EQEQEQEEQEEQELE----- | 3 | seq194 | EQEQEQEEQEEQELE |
| 14.1 | 4 | --EQEQEQEEQELEEVE----- | 3 | seq271 | EQEQEQEEQELEEVE |
| 14.1 | 5 | -----QEQEEQELEEVEEQE-- | 6 | seq339 | QEQEEQELEEVEEQE |
| 14.1 | 6 | -------QEEQELEEVEEQEQE | 8 | seq418 | QEEQELEEVEEQEQE |
| 15.1 | Consensus | VQEATAPGAVLLDAIDAESDKV | - | - | - |
| 15.1 | 1 | VQEATAPGAVLLDAI------- | 1 | seq281 | VQEATAPGAVLLDAI |
| 15.1 | 2 | --EATAPGAVLLDAIDA----- | 3 | seq243 | EATAPGAVLLDAIDA |
| 15.1 | 3 | ----TAPGAVLLDAIDAES--- | 5 | seq191 | TAPGAVLLDAIDAES |
| 15.1 | 4 | ------PGAVLLDAIDAESDK- | 7 | seq193 | PGAVLLDAIDAESDK |
| 15.1 | 5 | -------GAVLLDAIDAESDKV | 8 | seq160 | GAVLLDAIDAESDKV |
| 16.1 | Consensus | CSQHLPYIEQGMQLAEQ | - | - | - |
| 16.1 | 1 | CSQHLPYIEQGMQLA-- | 1 | seq1048 | CSQHLPYIEQGMQLA |
| 16.1 | 2 | --QHLPYIEQG------ | 3 | seq457 | QHLPYIEQG |
| 16.1 | 3 | ---HLPYIEQGMQLAEQ | 4 | seq402 | HLPYIEQGMQLAEQ |
| 16.1 | 4 | ----LPYIEQGMM---- | 5 | seq790 | LPYIEQGMM |
| 16.1 | 5 | -----PYIEQGMQL--- | 6 | seq665 | PYIEQGMQL |
| 17.1 | Consensus | NEQELLELDKWASLW | - | - | - |
| 17.1 | 1 | NEQELLELDKWASLW | 1 | seq928 | NEQELLELDKWASLW |
| 17.1 | 2 | ----LLELDKWA--- | 5 | seq915 | LLELDKWA |
| 17.1 | 3 | ------ELDKWNSL- | 7 | seq1041 | ELDKWNSL |
| 17.1 | 4 | ------ALDKWAS-- | 7 | seq489 | ALDKWAS |
| 17.2 | Singleton | ALDKWD | - | seq540 | ALDKWD |
| 18.1 | Consensus | EGKSEARGPSLEERIEEQGTRR | - | - | - |
| 18.1 | 1 | EGKSEARGPSLEERI------- | 1 | seq120 | EGKSEARGPSLEERI |
| 18.1 | 2 | --KSEARGPSLEERIEE----- | 3 | seq92 | KSEARGPSLEERIEE |
| 18.1 | 3 | ---SEARGPSLEERIEEQ---- | 4 | seq223 | SEARGPSLEERIEEQ |
| 18.1 | 4 | -----ARGPSLEERIEEQGT-- | 6 | seq477 | ARGPSLEERIEEQGT |
| 18.1 | 5 | -------GPSLEERIEEQGTRR | 8 | seq306 | GPSLEERIEEQGTRR |
| 19.1 | Consensus | GRTWAQPGYPWPLY | - | - | - |
| 19.1 | 1 | GRTWAQPGYPWP-- | 1 | seq857 | GRTWAQPGYPWP |
| 19.1 | 2 | ----AQPGYPWPLY | 5 | seq566 | AQPGYPWPLY |
| 19.1 | 3 | ----AQPGYPWP-- | 5 | seq906 | AQPGYPWP |
| 19.1 | 4 | -----QPGYPWPL- | 6 | seq924 | QPGYPWPL |
| 19.1 | 5 | ------PGYPWPLY | 7 | seq487 | PGYPWPLY |
| 20.1 | Consensus | XXEEEDDDMGFGLFD | - | - | - |
| 20.1 | 1 | AEEEEDDDMGFGLFD | 1 | seq689 | AEEEEDDDMGFGLFD |
| 20.1 | 2 | ESEESDDDMGFGLFD | 1 | seq925 | ESEESDDDMGFGLFD |
| 20.1 | 3 | --EEEDDDMGFGLFD | 3 | seq646 | EEEDDDMGFGLFD |
| 20.1 | 4 | ----EDDDMGFGLFD | 5 | seq794 | EDDDMGFGLFD |
| 20.1 | 5 | --------MGFGLFD | 9 | seq942 | MGFGLFD |
| 21.1 | Consensus | SPNVSVPSSSSTPLLY | - | - | - |
| 21.1 | 1 | SPNVSVPSSSSTPLLY | 1 | seq9 | SPNVSVPSSSSTPLLY |
| 21.1 | 2 | -PNVSVPSSSSTPLLY | 2 | seq5 | PNVSVPSSSSTPLLY |
| 21.1 | 3 | --NVSVPSSSSTPLLY | 3 | seq4 | NVSVPSSSSTPLLY |
| 21.1 | 4 | --NVSVPSPSSTPLLY | 3 | seq13 | NVSVPSPSSTPLLY |
| 22.1 | Consensus | AWLVHRQWFLDLPLPWL | - | - | - |
| 22.1 | 1 | AWLVHRQWFLDLPLPW- | 1 | seq458 | AWLVHRQWFLDLPLPW |
| 22.1 | 2 | ----HRQWFL------- | 5 | seq1132 | HRQWFL |
| 22.1 | 3 | -----RQWFLD------ | 6 | seq718 | RQWFLD |
| 22.1 | 4 | -----------LPLPWL | 12 | seq767 | LPLPWL |
| 23.1 | Consensus | TPIDTTIMA | - | - | - |
| 23.1 | 1 | TPIDTT--- | 1 | seq846 | TPIDTT |
| 23.1 | 2 | -PIDTTI-- | 2 | seq293 | PIDTTI |
| 23.1 | 3 | --IDTTIM- | 3 | seq113 | IDTTIM |
| 23.1 | 4 | ---DTTIMA | 4 | seq460 | DTTIMA |
| 24.1 | Consensus | TVTENDIRV | - | - | - |
| 24.1 | 1 | TVTEND--- | 1 | seq443 | TVTEND |
| 24.1 | 2 | -VTENDI-- | 2 | seq872 | VTENDI |
| 24.1 | 3 | --TENDIR- | 3 | seq867 | TENDIR |
| 24.1 | 4 | ---ENDIRV | 4 | seq1029 | ENDIRV |
| 25.1 | Consensus | HKXAIVTXTYDSEXQRXQ | - | - | - |
| 25.1 | 1 | HKSAIVTLTYDSEWQ--- | 1 | seq588 | HKSAIVTLTYDSEWQ |
| 25.1 | 2 | HKHAIVTVTYDSEEQ--- | 1 | seq776 | HKHAIVTVTYDSEEQ |
| 25.1 | 3 | ---AIVTLTYDSEWQRDQ | 4 | seq552 | AIVTLTYDSEWQRDQ |
| 25.1 | 4 | ---AIVTVTYDSEEQRQQ | 4 | seq431 | AIVTVTYDSEEQRQQ |
| 26.1 | Consensus | PPLLESWKDPDYVPP | - | - | - |
| 26.1 | 1 | PPLLESWKDPDYVPP | 1 | seq107 | PPLLESWKDPDYVPP |
| 26.1 | 2 | PPLLDRWKTPDYVPP | 1 | seq296 | PPLLDRWKTPDYVPP |
| 26.1 | 3 | -PLLESWKDPDYVPP | 2 | seq178 | PLLESWKDPDYVPP |
| 26.2 | Singleton | PLLDRWKAPDYVPP | - | seq422 | PLLDRWKAPDYVPP |
| 27.1 | Consensus | AAEGDDGDDGDEGGDGDEGEEGQE | - | - | - |
| 27.1 | 1 | AAEGDDGDDGDEGGDGD------- | 1 | seq259 | AAEGDDGDDGDEGGDGD |
| 27.1 | 2 | ---GDDGDDGDEGGDGDE------ | 4 | seq175 | GDDGDDGDEGGDGDE |
| 27.1 | 3 | ------GDDGDEGGDGDEGEEGQ- | 7 | seq73 | GDDGDEGGDGDEGEEGQ |
| 27.1 | 4 | ------------GGDGDEGEEGQE | 13 | seq108 | GGDGDEGEEGQE |
| 28.1 | Consensus | VEESIYQCCXLEPE | - | - | - |
| 28.1 | 1 | VEEEIYQCCNLEPE | 1 | seq235 | VEEEIYQCCNLEPE |
| 28.1 | 2 | --ESIYQC------ | 3 | seq866 | ESIYQC |
| 28.1 | 3 | ---SIYQCC----- | 4 | seq522 | SIYQCC |
| 28.1 | 4 | ----IYQCCD---- | 5 | seq1018 | IYQCCD |
| 29.1 | Consensus | EDDRRPLHPERVNVFDY | - | - | - |
| 29.1 | 1 | EDDRRPLHPERVNVF-- | 1 | seq679 | EDDRRPLHPERVNVF |
| 29.1 | 2 | --DRRPLHPERVNVFDY | 3 | seq173 | DRRPLHPERVNVFDY |
| 29.1 | 3 | ------LHPERVNVFDY | 7 | seq297 | LHPERVNVFDY |
| 29.1 | 4 | ------LHPERVNVFD- | 7 | seq502 | LHPERVNVFD |
| 30.1 | Consensus | GPGNGLGEKGDTSGPEGSGGSGP | - | - | - |
| 30.1 | 1 | GPGNGLGEKGDTSGPEG------ | 1 | seq559 | GPGNGLGEKGDTSGPEG |
| 30.1 | 2 | --GNGLGEKGDTSGPEGSG---- | 3 | seq564 | GNGLGEKGDTSGPEGSG |
| 30.1 | 3 | -----LGEKGDTSGPEGSGGSG- | 6 | seq702 | LGEKGDTSGPEGSGGSG |
| 30.1 | 4 | ------GEKGDTSGPEGSGGSGP | 7 | seq88 | GEKGDTSGPEGSGGSGP |
| 31.1 | Consensus | DEQEQQEEQEQQEEQEQELEEQ | - | - | - |
| 31.1 | 1 | DEQEQQEEQEQQEEQ------- | 1 | seq238 | DEQEQQEEQEQQEEQ |
| 31.1 | 2 | --QEQQEEQEQQEEQEQ----- | 3 | seq199 | QEQQEEQEQQEEQEQ |
| 31.1 | 3 | -----QEEQEQQEEQEQELE-- | 6 | seq239 | QEEQEQQEEQEQELE |
| 31.1 | 4 | -------EQEQQEEQEQELEEQ | 8 | seq237 | EQEQQEEQEQELEEQ |
| 32.1 | Consensus | QEGVQQEGAQQPAPATA | - | - | - |
| 32.1 | 1 | QEGVQQEGAQQPAP--- | 1 | seq633 | QEGVQQEGAQQPAP |
| 32.1 | 2 | -EGVQQEGAQQ------ | 2 | seq620 | EGVQQEGAQQ |
| 32.1 | 3 | ----QQEGAQQPAPATA | 5 | seq693 | QQEGAQQPAPATA |
| 32.1 | 4 | -----QEGAQQPAPA-- | 6 | seq711 | QEGAQQPAPA |
| 33.1 | Consensus | KVYVGNYDFWYQ | - | - | - |
| 33.1 | 1 | KVYVGNYDF--- | 1 | seq647 | KVYVGNYDF |
| 33.1 | 2 | -VYVGNYDFW-- | 2 | seq426 | VYVGNYDFW |
| 33.1 | 3 | --YVGNYDFWY- | 3 | seq221 | YVGNYDFWY |
| 33.1 | 4 | ---VGNYDFWYQ | 4 | seq591 | VGNYDFWYQ |
| 34.1 | Consensus | IKSLTERL | - | - | - |
| 34.1 | 1 | IKSLTE-- | 1 | seq249 | IKSLTE |
| 34.1 | 2 | -KSLTER- | 2 | seq248 | KSLTER |
| 34.1 | 3 | --SLTERL | 3 | seq151 | SLTERL |
| 35.1 | Consensus | TTFMLKYDENGTITDAVDCS | - | - | - |
| 35.1 | 1 | TTFMLKYDENGTITD----- | 1 | seq992 | TTFMLKYDENGTITD |
| 35.1 | 2 | ---MLKYDENGTITDAVD-- | 4 | seq1040 | MLKYDENGTITDAVD |
| 35.1 | 3 | -----KYDENGTITDAVDCS | 6 | seq837 | KYDENGTITDAVDCS |
| 36.1 | Consensus | PLDTPPAPXPPPFRLTPPAPAPPPFRLPLL | - | - | - |
| 36.1 | 1 | PLDTPPAPAPPPFRL--------------- | 1 | seq131 | PLDTPPAPAPPPFRL |
| 36.1 | 2 | -----PAPQPP------------------- | 6 | seq337 | PAPQPP |
| 36.1 | 3 | ---------------TPPAPAPPPFRLPLL | 16 | seq177 | TPPAPAPPPFRLPLL |
| 37.1 | Consensus | LKKDKEGXVSTLPXD | - | - | - |
| 37.1 | 1 | LKKDKEGKVSTLPLD | 1 | seq957 | LKKDKEGKVSTLPLD |
| 37.1 | 2 | -------VVSTLP-- | 8 | seq888 | VVSTLP |
| 37.1 | 3 | --------VSTLPQ- | 9 | seq565 | VSTLPQ |
| 38.1 | Consensus | DFTPGTEYTVEVR | - | - | - |
| 38.1 | 1 | DFTPGTEY----- | 1 | seq859 | DFTPGTEY |
| 38.1 | 2 | -FTPGTEYTVEVR | 2 | seq1116 | FTPGTEYTVEVR |
| 38.1 | 3 | --TPGTEYTV--- | 3 | seq468 | TPGTEYTV |
| 39.1 | Consensus | SSSTPLLYPSLALPA | - | - | - |
| 39.1 | 1 | SSSTPLLYPSLALPA | 1 | seq6 | SSSTPLLYPSLALPA |
| 39.1 | 2 | --STPLLYPSLA--- | 3 | seq8 | STPLLYPSLA |
| 39.1 | 3 | ----PLLYPS----- | 5 | seq53 | PLLYPS |
| 40.1 | Consensus | PPFSLSPVPTLGSRSRR | - | - | - |
| 40.1 | 1 | PPFSLSPVPTLGSRS-- | 1 | seq33 | PPFSLSPVPTLGSRS |
| 40.1 | 2 | PPFSLSPVPTL------ | 1 | seq20 | PPFSLSPVPTL |
| 40.1 | 3 | --FSLSPVPTLGSRSRR | 3 | seq176 | FSLSPVPTLGSRSRR |
| 41.1 | Consensus | XRLHQWINEDYPSP | - | - | - |
| 41.1 | 1 | RRLHQWINEDYPSP | 1 | seq421 | RRLHQWINEDYPSP |
| 41.1 | 2 | KRLHQWINED---- | 1 | seq404 | KRLHQWINED |
| 41.1 | 3 | --------EDYPSP | 9 | seq856 | EDYPSP |
| 42.1 | Consensus | KDTTSDYEKE | - | - | - |
| 42.1 | 1 | KDTTSDYE-- | 1 | seq576 | KDTTSDYE |
| 42.1 | 2 | -DTTSDYEK- | 2 | seq205 | DTTSDYEK |
| 42.1 | 3 | --TTSDYEKE | 3 | seq379 | TTSDYEKE |
| 43.1 | Consensus | RKPARLIVFPDL | - | - | - |
| 43.1 | 1 | RKPARLIVFPDL | 1 | seq1082 | RKPARLIVFPDL |
| 43.1 | 2 | -KPARLI----- | 2 | seq1025 | KPARLI |
| 43.1 | 3 | ------IVFPDL | 7 | seq869 | IVFPDL |
| 44.1 | Consensus | SQAQTASGLVSMF | - | - | - |
| 44.1 | 1 | SQAQTASGLV--- | 1 | seq808 | SQAQTASGLV |
| 44.1 | 2 | -QAQTASGLVS-- | 2 | seq850 | QAQTASGLVS |
| 44.1 | 3 | ---QTASGLVSMF | 4 | seq1002 | QTASGLVSMF |
| 45.1 | Consensus | VPESGEDREDARQ | - | - | - |
| 45.1 | 1 | VPESGEDREDARQ | 1 | seq423 | VPESGEDREDARQ |
| 45.1 | 2 | VPESGEDGEDARQ | 1 | seq1066 | VPESGEDGEDARQ |
| 45.1 | 3 | VPESGEDREDA-- | 1 | seq630 | VPESGEDREDA |
| 46.1 | Consensus | SKAALIEEGQR | - | - | - |
| 46.1 | 1 | SKAALIEEG-- | 1 | seq241 | SKAALIEEG |
| 46.1 | 2 | -KAALIEEGQ- | 2 | seq326 | KAALIEEGQ |
| 46.1 | 3 | --AALIEEGQR | 3 | seq879 | AALIEEGQR |
| 47.1 | Consensus | DAAVAEAEGG | - | - | - |
| 47.1 | 1 | DAAVAEAE-- | 1 | seq882 | DAAVAEAE |
| 47.1 | 2 | -AAVAEAEG- | 2 | seq883 | AAVAEAEG |
| 47.1 | 3 | --AVAEAEGG | 3 | seq730 | AVAEAEGG |
| 48.1 | Consensus | DSVKAASKETPPALNKC | - | - | - |
| 48.1 | 1 | DSVKAASKETPPAL--- | 1 | seq283 | DSVKAASKETPPAL |
| 48.1 | 2 | --VKAASKETPPALNK- | 3 | seq215 | VKAASKETPPALNK |
| 48.1 | 3 | ---KAASKETPPALNKC | 4 | seq126 | KAASKETPPALNKC |
| 49.1 | Consensus | LHPGSVNEFDF | - | - | - |
| 49.1 | 1 | LHPGSVNEFDF | 1 | seq261 | LHPGSVNEFDF |
| 49.1 | 2 | LHPGSVNEFD- | 1 | seq352 | LHPGSVNEFD |
| 49.1 | 3 | --PGSVNEFDF | 3 | seq365 | PGSVNEFDF |
| 50.1 | Consensus | AFASRGNHVSPTHYV | - | - | - |
| 50.1 | 1 | AFASRGNHDSPTHYV | 1 | seq44 | AFASRGNHDSPTHYV |
| 50.1 | 2 | AFASRGNHVAPTHYV | 1 | seq602 | AFASRGNHVAPTHYV |
| 50.1 | 3 | AFASRGNHVSPRHYV | 1 | seq1108 | AFASRGNHVSPRHYV |
| 51.1 | Consensus | QDEMKVIDDVQQLEKDKQQ | - | - | - |
| 51.1 | 1 | QDEMNVIDDVQQ------- | 1 | seq1098 | QDEMNVIDDVQQ |
| 51.1 | 2 | -DEMKVIDDVQQLEK---- | 2 | seq304 | DEMKVIDDVQQLEK |
| 51.1 | 3 | ---MKVIDDVQQLEKDKQQ | 4 | seq329 | MKVIDDVQQLEKDKQQ |
| 52.1 | Consensus | AKNEVFCV | - | - | - |
| 52.1 | 1 | AKNEVF-- | 1 | seq1081 | AKNEVF |
| 52.1 | 2 | -KNEVFC- | 2 | seq1135 | KNEVFC |
| 52.1 | 3 | --NEVFCV | 3 | seq751 | NEVFCV |
| 53.1 | Consensus | GVYLLPRRGPRLGVR | - | - | - |
| 53.1 | 1 | GVYLLPRRGPRLGVR | 1 | seq1059 | GVYLLPRRGPRLGVR |
| 53.1 | 2 | --YLLPRRGPRL--- | 3 | seq877 | YLLPRRGPRL |
| 53.1 | 3 | ----LPRRGPRL--- | 5 | seq1060 | LPRRGPRL |
| 54.1 | Consensus | ERKDRVEDAL | - | - | - |
| 54.1 | 1 | ERKDRVED-- | 1 | seq868 | ERKDRVED |
| 54.1 | 2 | -RKDRVEDA- | 2 | seq876 | RKDRVEDA |
| 54.1 | 3 | --KDRVEDAL | 3 | seq367 | KDRVEDAL |
| 55.1 | Consensus | EDDTTSEAAEGDVDPFPVL | - | - | - |
| 55.1 | 1 | EDDTTSEAAEGDVDP---- | 1 | seq610 | EDDTTSEAAEGDVDP |
| 55.1 | 2 | --DTTSEAAEGDVDPFP-- | 3 | seq94 | DTTSEAAEGDVDPFP |
| 55.1 | 3 | ----TSEAAEGDVDPFPVL | 5 | seq95 | TSEAAEGDVDPFPVL |
| 56.1 | Consensus | KEKMXLNEEEITTKG | - | - | - |
| 56.1 | 1 | KEKMILNEEEITTKG | 1 | seq988 | KEKMILNEEEITTKG |
| 56.1 | 2 | KEKMVLNEEEITTKG | 1 | seq935 | KEKMVLNEEEITTKG |
| 56.1 | 3 | KEKMLLNEEEITTKG | 1 | seq963 | KEKMLLNEEEITTKG |
| 57.1 | Consensus | ACSFSSIPNG | - | - | - |
| 57.1 | 1 | ACSFSSIP-- | 1 | seq1099 | ACSFSSIP |
| 57.1 | 2 | -CSFSSIPN- | 2 | seq621 | CSFSSIPN |
| 57.1 | 3 | --SFSSIPNG | 3 | seq495 | SFSSIPNG |
| 58.1 | Consensus | MAKVNIKPLEDKILV | - | - | - |
| 58.1 | 1 | MAKVKIKPLEDKILV | 1 | seq190 | MAKVKIKPLEDKILV |
| 58.1 | 2 | VAKVNIKPLEDKILV | 1 | seq211 | VAKVNIKPLEDKILV |
| 58.1 | 3 | MAKVNIKPLEDKILV | 1 | seq299 | MAKVNIKPLEDKILV |
| 59.1 | Consensus | GGSGGGFWGDRVDSQPFAI | - | - | - |
| 59.1 | 1 | GGSGGGFWGDRVDSQ---- | 1 | seq519 | GGSGGGFWGDRVDSQ |
| 59.1 | 2 | -GSGGGFWGDRVDSQP--- | 2 | seq699 | GSGGGFWGDRVDSQP |
| 59.1 | 3 | ----GGFWGDRVDSQPFAI | 5 | seq289 | GGFWGDRVDSQPFAI |
| 60.1 | Consensus | DPNANPNANPNANPNANP | - | - | - |
| 60.1 | 1 | DPNANPNVDPNANP---- | 1 | seq887 | DPNANPNVDPNANP |
| 60.1 | 2 | --NANPNANPNANPNANP | 3 | seq214 | NANPNANPNANPNANP |
| 60.1 | 3 | --NANPNANPNANP---- | 3 | seq325 | NANPNANPNANP |
| 61.1 | Consensus | NETIYNTTLKY | - | - | - |
| 61.1 | 1 | NETIYNTTLK- | 1 | seq170 | NETIYNTTLK |
| 61.1 | 2 | -ETIYNTTLKY | 2 | seq1 | ETIYNTTLKY |
| 62.1 | Consensus | PPYVEPTAPQVL | - | - | - |
| 62.1 | 1 | PPYVEPTAPQVL | 1 | seq14 | PPYVEPTAPQVL |
| 62.1 | 2 | -PYVEPTAPQVL | 2 | seq2 | PYVEPTAPQVL |
| 63.1 | Consensus | SLAQVKYTSASSI | - | - | - |
| 63.1 | 1 | SLAQVKYTSASSI | 1 | seq764 | SLAQVKYTSASSI |
| 63.1 | 2 | ----VKYTS---- | 5 | seq432 | VKYTS |
| 64.1 | Consensus | GPEKAFREL | - | - | - |
| 64.1 | 1 | GPEKAFRE- | 1 | seq1006 | GPEKAFRE |
| 64.1 | 2 | -PEKAFREL | 2 | seq752 | PEKAFREL |
| 65.1 | Consensus | AMDPIYDEPTTTTSVPL | - | - | - |
| 65.1 | 1 | AMDPIYDEPTTTTSV-- | 1 | seq11 | AMDPIYDEPTTTTSV |
| 65.1 | 2 | --DPIYDEPTTTTSVPL | 3 | seq10 | DPIYDEPTTTTSVPL |
| 66.1 | Consensus | ETLVTFKN | - | - | - |
| 66.1 | 1 | ETLVTFKN | 1 | seq1008 | ETLVTFKN |
| 66.1 | 2 | ETLVTF-- | 1 | seq964 | ETLVTF |
| 67.1 | Consensus | RFQPFQQFGRDVSDFTD | - | - | - |
| 67.1 | 1 | RFQPFQQFGRDVSDF-- | 1 | seq317 | RFQPFQQFGRDVSDF |
| 67.1 | 2 | --QPFQQFGRDVSDFTD | 3 | seq273 | QPFQQFGRDVSDFTD |
| 68.1 | Consensus | KLRPFERDISNVPFSPD | - | - | - |
| 68.1 | 1 | KLRPFERDISNV----- | 1 | seq1055 | KLRPFERDISNV |
| 68.1 | 2 | --RPFERDISNVPFSPD | 3 | seq909 | RPFERDISNVPFSPD |
| 69.1 | Consensus | PPSSPTHDPPDSDPQI | - | - | - |
| 69.1 | 1 | PPSSPTHDPPDSDPQI | 1 | seq15 | PPSSPTHDPPDSDPQI |
| 69.1 | 2 | PPSSPTHDPPDSDP-- | 1 | seq71 | PPSSPTHDPPDSDP |
| 70.1 | Consensus | SPGGLEPPSEKHFRETEV | - | - | - |
| 70.1 | 1 | SPGGLEPPSEKHFRE--- | 1 | seq16 | SPGGLEPPSEKHFRE |
| 70.1 | 2 | ---GLEPPSEKHFRETEV | 4 | seq30 | GLEPPSEKHFRETEV |
| 71.1 | Consensus | TPSPAPSRPFSVLRANDV | - | - | - |
| 71.1 | 1 | TPSPAPSRPFSVLRA--- | 1 | seq761 | TPSPAPSRPFSVLRA |
| 71.1 | 2 | ---PAPSRPFSVLRANDV | 4 | seq801 | PAPSRPFSVLRANDV |
| 72.1 | Consensus | PEEFEGAGDGEPPEDDDS | - | - | - |
| 72.1 | 1 | PEEFEGAGDGEPPED--- | 1 | seq21 | PEEFEGAGDGEPPED |
| 72.1 | 2 | ---FEGAGDGEPPEDDDS | 4 | seq25 | FEGAGDGEPPEDDDS |
| 73.1 | Consensus | EADYFEYHQEGGPDGEPDVP | - | - | - |
| 73.1 | 1 | EADYFEYHQEGGPDGEP--- | 1 | seq55 | EADYFEYHQEGGPDGEP |
| 73.1 | 2 | ---YFEYHQEGGPDGEPDVP | 4 | seq91 | YFEYHQEGGPDGEPDVP |
| 74.1 | Consensus | QPELAPEDPEDS | - | - | - |
| 74.1 | 1 | QPELAPEDPED- | 1 | seq266 | QPELAPEDPED |
| 74.1 | 2 | --ELAPEDPEDS | 3 | seq242 | ELAPEDPEDS |
| 75.1 | Consensus | AIPTNFSISITTEVM | - | - | - |
| 75.1 | 1 | AIPTNFSISITTEVM | 1 | seq670 | AIPTNFSISITTEVM |
| 75.1 | 2 | ----NFSISI----- | 5 | seq1030 | NFSISI |
| 76.1 | Consensus | SKTVGTAEEKLKKK | - | - | - |
| 76.1 | 1 | SKTVGTAEEKLKKK | 1 | seq673 | SKTVGTAEEKLKKK |
| 76.1 | 2 | --TVGTAEEKLK-- | 3 | seq1004 | TVGTAEEKLK |
| 77.1 | Consensus | PAGRNNDGSSAP | - | - | - |
| 77.1 | 1 | PAGRNNDGSSAP | 1 | seq1122 | PAGRNNDGSSAP |
| 77.1 | 2 | ---RNNDGSSAP | 4 | seq874 | RNNDGSSAP |
| 78.1 | Consensus | FAIPYIHPTNPFAPDVTA | - | - | - |
| 78.1 | 1 | FAIPYIHPTNPFAPD--- | 1 | seq35 | FAIPYIHPTNPFAPD |
| 78.1 | 2 | ---PYIHPTNPFAPDVTA | 4 | seq694 | PYIHPTNPFAPDVTA |
| 79.1 | Consensus | HMTNYLEQPVSNDLSNC | - | - | - |
| 79.1 | 1 | HMTNYLEQPVSNDLS-- | 1 | seq649 | HMTNYLEQPVSNDLS |
| 79.1 | 2 | --TNYLEQPVSNDLSNC | 3 | seq377 | TNYLEQPVSNDLSNC |
| 80.1 | Consensus | VRYPHYSLIKPESSL | - | - | - |
| 80.1 | 1 | VRYPHYSLIKPESSL | 1 | seq557 | VRYPHYSLIKPESSL |
| 80.1 | 2 | -RYPHYSLIKPESSL | 2 | seq648 | RYPHYSLIKPESSL |
| 81.1 | Consensus | YQPIDVVRDLPSGFNTL | - | - | - |
| 81.1 | 1 | YQPIDVVRDLPSGFN-- | 1 | seq1026 | YQPIDVVRDLPSGFN |
| 81.1 | 2 | --PIDVVRDLPSGFNTL | 3 | seq864 | PIDVVRDLPSGFNTL |
| 82.1 | Consensus | EXSSSDKP | - | - | - |
| 82.1 | 1 | EASSSDKP | 1 | seq257 | EASSSDKP |
| 82.1 | 2 | ESSSSDKP | 1 | seq264 | ESSSSDKP |
| 83.1 | Consensus | RGNHXXPTHYVXESD | - | - | - |
| 83.1 | 1 | RGNHDSPTHYVPESD | 1 | seq130 | RGNHDSPTHYVPESD |
| 83.1 | 2 | RGNHVAPTHYVTESD | 1 | seq721 | RGNHVAPTHYVTESD |
| 84.1 | Consensus | DTRCFDS | - | - | - |
| 84.1 | 1 | DTRCFD- | 1 | seq471 | DTRCFD |
| 84.1 | 2 | -TRCFDS | 2 | seq884 | TRCFDS |
| 85.1 | Consensus | AGCLIGAEHVDTSYECD | - | - | - |
| 85.1 | 1 | AGCLIGAEHVDTSYE-- | 1 | seq180 | AGCLIGAEHVDTSYE |
| 85.1 | 2 | --CLIGAEHVDTSYECD | 3 | seq182 | CLIGAEHVDTSYECD |
| 86.1 | Consensus | FTDSVRDPKTSEILDIS | - | - | - |
| 86.1 | 1 | FTDSVRDPKTSEILD-- | 1 | seq707 | FTDSVRDPKTSEILD |
| 86.1 | 2 | --DSVRDPKTSEILDIS | 3 | seq464 | DSVRDPKTSEILDIS |
| 87.1 | Consensus | YISDAFSLDVSEKSGNF | - | - | - |
| 87.1 | 1 | YISDAFSLDVSEKSG-- | 1 | seq703 | YISDAFSLDVSEKSG |
| 87.1 | 2 | --SDAFSLDVSEKSGNF | 3 | seq417 | SDAFSLDVSEKSGNF |
| 88.1 | Consensus | TVYDPLQPELDSFKEEL | - | - | - |
| 88.1 | 1 | TVYDPLQPELDSFKE-- | 1 | seq849 | TVYDPLQPELDSFKE |
| 88.1 | 2 | --YDPLQPELDSFKEEL | 3 | seq586 | YDPLQPELDSFKEEL |
| 89.1 | Consensus | DSKNFNFEV | - | - | - |
| 89.1 | 1 | DSKNFNFE- | 1 | seq683 | DSKNFNFE |
| 89.1 | 2 | -SKNFNFEV | 2 | seq979 | SKNFNFEV |
| 90.1 | Consensus | CTPPALNCYWPLNDYGF | - | - | - |
| 90.1 | 1 | CTPPALNCYWPLNDY-- | 1 | seq138 | CTPPALNCYWPLNDY |
| 90.1 | 2 | --PPALNCYWPLNDYGF | 3 | seq515 | PPALNCYWPLNDYGF |
| 91.1 | Consensus | VIGIINNTVYDPLQPEL | - | - | - |
| 91.1 | 1 | VIGIINNTVYDPLQP-- | 1 | seq697 | VIGIINNTVYDPLQP |
| 91.1 | 2 | --GIINNTVYDPLQPEL | 3 | seq159 | GIINNTVYDPLQPEL |
| 92.1 | Consensus | KKPNRQGLGYYSPSYNDP | - | - | - |
| 92.1 | 1 | KKPNRQGLGYYSPSY--- | 1 | seq916 | KKPNRQGLGYYSPSY |
| 92.1 | 2 | ---NRQGLGYYSPSYNDP | 4 | seq398 | NRQGLGYYSPSYNDP |
| 93.1 | Consensus | RPXXDFDQGWGPIXY | - | - | - |
| 93.1 | 1 | RPIDDFDQGWGPITY | 1 | seq355 | RPIDDFDQGWGPITY |
| 93.1 | 2 | RPLIDFDQGWGPISY | 1 | seq533 | RPLIDFDQGWGPISY |
| 94.1 | Consensus | QLPQGTTLPKGFYAEGSR | - | - | - |
| 94.1 | 1 | QLPQGTTLPKGFYAE--- | 1 | seq695 | QLPQGTTLPKGFYAE |
| 94.1 | 2 | ---QGTTLPKGFYAEGSR | 4 | seq726 | QGTTLPKGFYAEGSR |
| 95.1 | Consensus | GCKGTHGGTGAGAGAGGAGA | - | - | - |
| 95.1 | 1 | GCKGTHGGTGAGAGAGG--- | 1 | seq217 | GCKGTHGGTGAGAGAGG |
| 95.1 | 2 | ---GTHGGTGAGAGAGGAGA | 4 | seq77 | GTHGGTGAGAGAGGAGA |
| 96.1 | Consensus | AEPKSAEPKPAEPKS | - | - | - |
| 96.1 | 1 | AEPKSAEPKPAEPKS | 1 | seq320 | AEPKSAEPKPAEPKS |
| 96.1 | 2 | -----AEPKPAEPKS | 6 | seq357 | AEPKPAEPKS |
| 97.1 | Consensus | SASDQPTQYEEEMTDYQK | - | - | - |
| 97.1 | 1 | SASDQPTQYEEEMTD--- | 1 | seq68 | SASDQPTQYEEEMTD |
| 97.1 | 2 | ---DQPTQYEEEMTDYQK | 4 | seq75 | DQPTQYEEEMTDYQK |
| 98.1 | Consensus | QFGRDVSDFTDSVRD | - | - | - |
| 98.1 | 1 | QFGRDVSDFTDSVRD | 1 | seq600 | QFGRDVSDFTDSVRD |
| 98.1 | 2 | -----VSDFTDSV-- | 6 | seq658 | VSDFTDSV |
| 99.1 | Consensus | VTNDCSNSSIVYEAADVI | - | - | - |
| 99.1 | 1 | VTNDCSNSSIVYEAA--- | 1 | seq338 | VTNDCSNSSIVYEAA |
| 99.1 | 2 | ---DCSNSSIVYEAADVI | 4 | seq78 | DCSNSSIVYEAADVI |
| 100.1 | Consensus | MSDEGPGTGPGNGLGEKGDT | - | - | - |
| 100.1 | 1 | MSDEGPGTGPGNGLGEK--- | 1 | seq1107 | MSDEGPGTGPGNGLGEK |
| 100.1 | 2 | ---EGPGTGPGNGLGEKGDT | 4 | seq282 | EGPGTGPGNGLGEKGDT |
| 101.1 | Consensus | EGDVDPFPVLANEGKSE | - | - | - |
| 101.1 | 1 | EGDVDPFPVLANEGK-- | 1 | seq150 | EGDVDPFPVLANEGK |
| 101.1 | 2 | --DVDPFPVLANEGKSE | 3 | seq164 | DVDPFPVLANEGKSE |
| 102.1 | Consensus | TYXWGANDTDVFVL | - | - | - |
| 102.1 | 1 | TYNWGANDTDVFVL | 1 | seq686 | TYNWGANDTDVFVL |
| 102.1 | 2 | --SWGANDTDVFVL | 3 | seq1056 | SWGANDTDVFVL |
| 103.1 | Consensus | GTIVIRVQYEG | - | - | - |
| 103.1 | 1 | GTIVIRVQYEG | 1 | seq623 | GTIVIRVQYEG |
| 103.1 | 2 | ----IRVQYE- | 5 | seq555 | IRVQYE |
| 104.1 | Consensus | ALVPDKEVLY | - | - | - |
| 104.1 | 1 | ALVPDKEVL- | 1 | seq940 | ALVPDKEVL |
| 104.1 | 2 | -LVPDKEVLY | 2 | seq455 | LVPDKEVLY |
| 105.1 | Consensus | TSHPIEEIPLDTFVX | - | - | - |
| 105.1 | 1 | TSHPIEEIPLDTFVI | 1 | seq262 | TSHPIEEIPLDTFVI |
| 105.1 | 2 | TSHPIEEIPLDTFVV | 1 | seq268 | TSHPIEEIPLDTFVV |
| 106.1 | Consensus | STHNYEEIXMDTFIVST | - | - | - |
| 106.1 | 1 | STHNYEEIRMDTFIV-- | 1 | seq318 | STHNYEEIRMDTFIV |
| 106.1 | 2 | --HNYEEIPMDTFIVST | 3 | seq650 | HNYEEIPMDTFIVST |
| 107.1 | Consensus | KSIVAYTMSLGADSSIA | - | - | - |
| 107.1 | 1 | KSIVAYTMSLGADSS-- | 1 | seq845 | KSIVAYTMSLGADSS |
| 107.1 | 2 | --IVAYTMSLGADSSIA | 3 | seq341 | IVAYTMSLGADSSIA |
| 108.1 | Consensus | DDIVGYIMHGISTINTE | - | - | - |
| 108.1 | 1 | DDIVGYIMHGISTIN-- | 1 | seq782 | DDIVGYIMHGISTIN |
| 108.1 | 2 | --IVGYIMHGISTINTE | 3 | seq687 | IVGYIMHGISTINTE |
| 109.1 | Consensus | QQQGQTVTKKSAAEASKK | - | - | - |
| 109.1 | 1 | QQQGQTVTKKSAAEA--- | 1 | seq543 | QQQGQTVTKKSAAEA |
| 109.1 | 2 | ---GQTVTKKSAAEASKK | 4 | seq115 | GQTVTKKSAAEASKK |
| 110.1 | Consensus | GYYYPSDPNTFYAPPYS | - | - | - |
| 110.1 | 1 | GYYYPSDPNTFYAPP-- | 1 | seq609 | GYYYPSDPNTFYAPP |
| 110.1 | 2 | --YYPSDPNTFYAPPYS | 3 | seq563 | YYPSDPNTFYAPPYS |
| 111.1 | Consensus | IAPGQTGVIADYNYKLP | - | - | - |
| 111.1 | 1 | IAPGQTGVIADYNYK-- | 1 | seq616 | IAPGQTGVIADYNYK |
| 111.1 | 2 | --PGQTGVIADYNYKLP | 3 | seq975 | PGQTGVIADYNYKLP |
| 112.1 | Consensus | ELTEXQQXGDEPL | - | - | - |
| 112.1 | 1 | ELTEQQQTGDEPL | 1 | seq136 | ELTEQQQTGDEPL |
| 112.1 | 2 | ELTEEQQRGDEPL | 1 | seq316 | ELTEEQQRGDEPL |
| 113.1 | Consensus | SLNEEQD | - | - | - |
| 113.1 | 1 | SLNEEQ- | 1 | seq747 | SLNEEQ |
| 113.1 | 2 | -LNEEQD | 2 | seq724 | LNEEQD |
| 114.1 | Consensus | LVYQTEKFV | - | - | - |
| 114.1 | 1 | LVYQTEKF- | 1 | seq324 | LVYQTEKF |
| 114.1 | 2 | -VYQTEKFV | 2 | seq716 | VYQTEKFV |
| 115.1 | Consensus | SVENAQQDKGIAIPHDID | - | - | - |
| 115.1 | 1 | SVENAQQDKGIAIPH--- | 1 | seq277 | SVENAQQDKGIAIPH |
| 115.1 | 2 | ---NAQQDKGIAIPHDID | 4 | seq179 | NAQQDKGIAIPHDID |
| 116.1 | Consensus | KGLRRDLDASREAKKQ | - | - | - |
| 116.1 | 1 | KGLRRDLDASREAKKQ | 1 | seq408 | KGLRRDLDASREAKKQ |
| 116.1 | 2 | ---RRDLDASREAKK- | 4 | seq236 | RRDLDASREAKK |
| 117.1 | Consensus | AMLTAFFLRRTGRRSPQ | - | - | - |
| 117.1 | 1 | AMLTAFFLRRTGRRS-- | 1 | seq652 | AMLTAFFLRRTGRRS |
| 117.1 | 2 | --LTAFFLRRTGRRSPQ | 3 | seq1028 | LTAFFLRRTGRRSPQ |
| 118.1 | Consensus | GGESSEELSESSFFNLIT | - | - | - |
| 118.1 | 1 | GGESSEELSESSFFN--- | 1 | seq435 | GGESSEELSESSFFN |
| 118.1 | 2 | ---SSEELSESSFFNLIT | 4 | seq709 | SSEELSESSFFNLIT |
| 119.1 | Consensus | TDWDVKGGGSPLYRHGD | - | - | - |
| 119.1 | 1 | TDWDVKGGGSPLYRH-- | 1 | seq279 | TDWDVKGGGSPLYRH |
| 119.1 | 2 | --WDVKGGGSPLYRHGD | 3 | seq684 | WDVKGGGSPLYRHGD |
| 120.1 | Consensus | WARRNGHNVSTRGRIPAD | - | - | - |
| 120.1 | 1 | WARRNGHNVSTRGRI--- | 1 | seq1138 | WARRNGHNVSTRGRI |
| 120.1 | 2 | ---RNGHNVSTRGRIPAD | 4 | seq220 | RNGHNVSTRGRIPAD |
| 121.1 | Consensus | NAFNCTFEYISDAFSLD | - | - | - |
| 121.1 | 1 | NAFNCTFEYISDAFS-- | 1 | seq852 | NAFNCTFEYISDAFS |
| 121.1 | 2 | --FNCTFEYISDAFSLD | 3 | seq315 | FNCTFEYISDAFSLD |
| 122.1 | Consensus | SSEVAVLYQDVNCTDV | - | - | - |
| 122.1 | 1 | SSEVAVLYQDVNCTDV | 1 | seq401 | SSEVAVLYQDVNCTDV |
| 122.1 | 2 | SSEVAVLYQDVNCTD- | 1 | seq681 | SSEVAVLYQDVNCTD |
| 123.1 | Consensus | TLPFNWTHCFDPQIQ | - | - | - |
| 123.1 | 1 | TLPFNWTHCFDPQIQ | 1 | seq446 | TLPFNWTHCFDPQIQ |
| 123.1 | 2 | -LPFNWTHCFDPQ-- | 2 | seq1023 | LPFNWTHCFDPQ |
| 124.1 | Consensus | IMSGEKPXXEDVVN | - | - | - |
| 124.1 | 1 | IMSGEKPTVEDVVN | 1 | seq562 | IMSGEKPTVEDVVN |
| 124.1 | 2 | IMSGEKPSMEDVVN | 1 | seq704 | IMSGEKPSMEDVVN |
| 125.1 | Consensus | QALNTLVKQLSSNFGAI | - | - | - |
| 125.1 | 1 | QALNTLVKQLSSNFG-- | 1 | seq1009 | QALNTLVKQLSSNFG |
| 125.1 | 2 | --LNTLVKQLSSNFGAI | 3 | seq696 | LNTLVKQLSSNFGAI |
| 126.1 | Consensus | PISYANGSGXDXRPY | - | - | - |
| 126.1 | 1 | PISYANGSGPDQRPY | 1 | seq659 | PISYANGSGPDQRPY |
| 126.1 | 2 | -ISYANGSGLDERPY | 2 | seq946 | ISYANGSGLDERPY |
| 127.1 | Consensus | KKSAAEASKKPRQKRTA | - | - | - |
| 127.1 | 1 | KKSAAEASKKPRQKR-- | 1 | seq161 | KKSAAEASKKPRQKR |
| 127.1 | 2 | --SAAEASKKPRQKRTA | 3 | seq656 | SAAEASKKPRQKRTA |
| 128.1 | Consensus | CFDPQIQAIVSSPCHNS | - | - | - |
| 128.1 | 1 | CFDPQIQAIVSSPCHN- | 1 | seq162 | CFDPQIQAIVSSPCHN |
| 128.1 | 2 | -FDPQIQAIVSSPCHNS | 2 | seq254 | FDPQIQAIVSSPCHNS |
| 129.1 | Consensus | ADQPXNHQALAEPV | - | - | - |
| 129.1 | 1 | ADQPENHQALAEPV | 1 | seq212 | ADQPENHQALAEPV |
| 129.1 | 2 | ADQPGNHQALAEPV | 1 | seq821 | ADQPGNHQALAEPV |
| 130.1 | Consensus | IADYNYKLPDDFMGCVL | - | - | - |
| 130.1 | 1 | IADYNYKLPDDFMGC-- | 1 | seq628 | IADYNYKLPDDFMGC |
| 130.1 | 2 | --DYNYKLPDDFMGCVL | 3 | seq598 | DYNYKLPDDFMGCVL |
| 131.1 | Consensus | DSXSXSDSDSDSDSD | - | - | - |
| 131.1 | 1 | DSESDSDSDSDSDSD | 1 | seq198 | DSESDSDSDSDSDSD |
| 131.1 | 2 | DSDSESDSDSDSDSD | 1 | seq204 | DSDSESDSDSDSDSD |
| 132.1 | Consensus | NFNFEVEVV | - | - | - |
| 132.1 | 1 | NFNFEVEV- | 1 | seq731 | NFNFEVEV |
| 132.1 | 2 | -FNFEVEVV | 2 | seq1001 | FNFEVEVV |
| 133.1 | Consensus | NNKSQSVIIINNSTNVV | - | - | - |
| 133.1 | 1 | NNKSQSVIIINNSTN-- | 1 | seq364 | NNKSQSVIIINNSTN |
| 133.1 | 2 | --KSQSVIIINNSTNVV | 3 | seq224 | KSQSVIIINNSTNVV |
| 134.1 | Consensus | KLNDLCFSNVYADSFVV | - | - | - |
| 134.1 | 1 | KLNDLCFSNVYADSF-- | 1 | seq226 | KLNDLCFSNVYADSF |
| 134.1 | 2 | --NDLCFSNVYADSFVV | 3 | seq653 | NDLCFSNVYADSFVV |
| 135.1 | Consensus | PPPYAPNPGPPPPYTGA | - | - | - |
| 135.1 | 1 | PPPYAPNPGPPPPYT-- | 1 | seq228 | PPPYAPNPGPPPPYT |
| 135.1 | 2 | --PYAPNPGPPPPYTGA | 3 | seq847 | PYAPNPGPPPPYTGA |
| 136.1 | Consensus | DRCTTFDDVQAPNYTQH | - | - | - |
| 136.1 | 1 | DRCTTFDDVQAPNYT-- | 1 | seq251 | DRCTTFDDVQAPNYT |
| 136.1 | 2 | --CTTFDDVQAPNYTQH | 3 | seq245 | CTTFDDVQAPNYTQH |
| 137.1 | Consensus | KQXGENFAYLTAYQA | - | - | - |
| 137.1 | 1 | KQSGENFAYLTAYQA | 1 | seq627 | KQSGENFAYLTAYQA |
| 137.1 | 2 | KQGGENFAYLTAYQA | 1 | seq676 | KQGGENFAYLTAYQA |
| 138.1 | Consensus | KPQEEKEKITKEILNGK | - | - | - |
| 138.1 | 1 | KPQEEKEKITKEILN-- | 1 | seq444 | KPQEEKEKITKEILN |
| 138.1 | 2 | --QEEKEKITKEILNGK | 3 | seq439 | QEEKEKITKEILNGK |
| 139.1 | Consensus | NGTITVEELKQLLEQWNL | - | - | - |
| 139.1 | 1 | NGTITVEELKQLLEQ--- | 1 | seq512 | NGTITVEELKQLLEQ |
| 139.1 | 2 | ---ITVEELKQLLEQWNL | 4 | seq692 | ITVEELKQLLEQWNL |
| 140.1 | Consensus | XSAEGDLVGWPSPP | - | - | - |
| 140.1 | 1 | SSAEGDLVGWPSPP | 1 | seq250 | SSAEGDLVGWPSPP |
| 140.1 | 2 | TSAEGDLVGWPSPP | 1 | seq290 | TSAEGDLVGWPSPP |
| 141.1 | Consensus | LSIVMPVGGQSSFYSDWY | - | - | - |
| 141.1 | 1 | LSIVMPVGGQSSFYS--- | 1 | seq319 | LSIVMPVGGQSSFYS |
| 141.1 | 2 | ---VMPVGGQSSFYSDWY | 4 | seq276 | VMPVGGQSSFYSDWY |
| 142.1 | Consensus | KMVQVVYD | - | - | - |
| 142.1 | 1 | KMVQVVYD | 1 | seq996 | KMVQVVYD |
| 142.1 | 2 | -MVQVVYD | 2 | seq811 | MVQVVYD |
| 143.1 | Consensus | KEEKEEKEEKEEKEKEKE | - | - | - |
| 143.1 | 1 | KEEKEEKEEKEEKEK--- | 1 | seq286 | KEEKEEKEEKEEKEK |
| 143.1 | 2 | ---KEEKEEKEEKEKEKE | 4 | seq333 | KEEKEEKEEKEKEKE |
| 144.1 | Consensus | WGVTTSLSYF | - | - | - |
| 144.1 | 1 | WGVTTSLSY- | 1 | seq295 | WGVTTSLSY |
| 144.1 | 2 | -GVTTSLSYF | 2 | seq500 | GVTTSLSYF |
| 145.1 | Consensus | KCNEKHDEEFCDMLRL | - | - | - |
| 145.1 | 1 | KCNEKHDEEFCDMLRL | 1 | seq497 | KCNEKHDEEFCDMLRL |
| 145.1 | 2 | --NEKHDEEF------ | 3 | seq788 | NEKHDEEF |
| 146.1 | Consensus | EERIEEQGTRRRYSSVQ | - | - | - |
| 146.1 | 1 | EERIEEQGTRRRYSS-- | 1 | seq1102 | EERIEEQGTRRRYSS |
| 146.1 | 2 | --RIEEQGTRRRYSSVQ | 3 | seq424 | RIEEQGTRRRYSSVQ |
| 147.1 | Consensus | KEQFEFALTAVAEEVC | - | - | - |
| 147.1 | 1 | KEQFEFALTAVAEEVC | 1 | seq574 | KEQFEFALTAVAEEVC |
| 147.1 | 2 | KEQFEFALTAVAEEV- | 1 | seq466 | KEQFEFALTAVAEEV |
| 148.1 | Consensus | NLVLNRDVSFQDLSD | - | - | - |
| 148.1 | 1 | NLVLNRDVSFQDL-- | 1 | seq720 | NLVLNRDVSFQDL |
| 148.1 | 2 | --VLNRDVSFQDLSD | 3 | seq372 | VLNRDVSFQDLSD |
| 149.1 | Consensus | AQYAAQNRRGLDLLFWE | - | - | - |
| 149.1 | 1 | AQYAAQNRRGLDLLFW- | 1 | seq593 | AQYAAQNRRGLDLLFW |
| 149.1 | 2 | --YAAQNRRGLDLLFWE | 3 | seq397 | YAAQNRRGLDLLFWE |
| 150.1 | Consensus | XXNDDDNDDXNDDNN | - | - | - |
| 150.1 | 1 | DDNDDDNDDNNDDNN | 1 | seq409 | DDNDDDNDDNNDDNN |
| 150.1 | 2 | YNNDDDNDDDNDDNN | 1 | seq606 | YNNDDDNDDDNDDNN |
| 151.1 | Consensus | RRPQKRPSCIGCKGTHGGTG | - | - | - |
| 151.1 | 1 | RRPQKRPSCIGCKGTHG--- | 1 | seq505 | RRPQKRPSCIGCKGTHG |
| 151.1 | 2 | ---QKRPSCIGCKGTHGGTG | 4 | seq475 | QKRPSCIGCKGTHGGTG |
| 152.1 | Consensus | DGDGAAAMRYTEARMTK | - | - | - |
| 152.1 | 1 | DGDGAAAMRYTEARM-- | 1 | seq833 | DGDGAAAMRYTEARM |
| 152.1 | 2 | --DGAAAMRYTEARMTK | 3 | seq605 | DGAAAMRYTEARMTK |
| 153.1 | Consensus | SAAIGLSMAGSSAMILAA | - | - | - |
| 153.1 | 1 | SAAIGLSMAGSSAMI--- | 1 | seq1012 | SAAIGLSMAGSSAMI |
| 153.1 | 2 | ---IGLSMAGSSAMILAA | 4 | seq531 | IGLSMAGSSAMILAA |
| 154.1 | Consensus | TSGTSGXSGXSGTSX | - | - | - |
| 154.1 | 1 | TSGTSGTSGTSGTSG | 1 | seq585 | TSGTSGTSGTSGTSG |
| 154.1 | 2 | TSGTSGPSGPSGTSP | 1 | seq931 | TSGTSGPSGPSGTSP |
| 155.1 | Singleton | NEITERYFKLAENYY | - | seq526 | NEITERYFKLAENYY |
| 156.1 | Singleton | TERLYI | - | seq534 | TERLYI |
| 157.1 | Singleton | PKEITVATSRTLSYY | - | seq671 | PKEITVATSRTLSYY |
| 158.1 | Singleton | CIKTLMTNVETYFK | - | seq717 | CIKTLMTNVETYFK |
| 159.1 | Singleton | KNTLTPLEELYPT | - | seq905 | KNTLTPLEELYPT |
| 160.1 | Singleton | EAHVPPPYVEPTTTQ | - | seq3 | EAHVPPPYVEPTTTQ |
| 161.1 | Singleton | YEVEKPLEPAPVAPS | - | seq524 | YEVEKPLEPAPVAPS |
| 162.1 | Singleton | GSQLPCEPEPDVAVL | - | seq603 | GSQLPCEPEPDVAVL |
| 163.1 | Singleton | PCEPEPDVAVLTSML | - | seq626 | PCEPEPDVAVLTSML |
| 164.1 | Singleton | QRPTPTQVLEEDPSV | - | seq727 | QRPTPTQVLEEDPSV |
| 165.1 | Singleton | VPLPKDQRPTPTQVLE | - | seq797 | VPLPKDQRPTPTQVLE |
| 166.1 | Singleton | GYEPPTVLGCALPPT | - | seq1062 | GYEPPTVLGCALPPT |
| 167.1 | Singleton | SSATK | - | seq719 | SSATK |
| 168.1 | Singleton | YPYDVPDYAG | - | seq125 | YPYDVPDYAG |
| 169.1 | Singleton | KEFED | - | seq447 | KEFED |
| 170.1 | Singleton | YFEPGPT | - | seq640 | YFEPGPT |
| 171.1 | Singleton | FPSDEF | - | seq734 | FPSDEF |
| 172.1 | Singleton | GGAVP | - | seq855 | GGAVP |
| 173.1 | Singleton | APGGGF | - | seq875 | APGGGF |
| 174.1 | Singleton | GQRVEF | - | seq1007 | GQRVEF |
| 175.1 | Singleton | DGEGAP | - | seq1067 | DGEGAP |
| 176.1 | Singleton | PHSALALLEDTMDYP | - | seq54 | PHSALALLEDTMDYP |
| 177.1 | Singleton | DSGSRTPPDELALS | - | seq509 | DSGSRTPPDELALS |
| 178.1 | Singleton | PSTLRSLRKRRLSSP | - | seq579 | PSTLRSLRKRRLSSP |
| 179.1 | Singleton | PSTLQTPGSSSGASL | - | seq634 | PSTLQTPGSSSGASL |
| 180.1 | Singleton | ASAFSRPGLPVEYLQ | - | seq970 | ASAFSRPGLPVEYLQ |
| 181.1 | Singleton | ALLRCIPALDSLTPA | - | seq1118 | ALLRCIPALDSLTPA |
| 182.1 | Singleton | NLIDTS | - | seq156 | NLIDTS |
| 183.1 | Singleton | DPPFSP | - | seq780 | DPPFSP |
| 184.1 | Singleton | DSTVTE | - | seq789 | DSTVTE |
| 185.1 | Singleton | SLQPED | - | seq1037 | SLQPED |
| 186.1 | Singleton | GSGPRHRDGVRRPQKRP | - | seq433 | GSGPRHRDGVRRPQKRP |
| 187.1 | Singleton | KQHVRDGRKDSLDGF | - | seq536 | KQHVRDGRKDSLDGF |
| 188.1 | Singleton | HGKEELRFPRGQGVPI | - | seq560 | HGKEELRFPRGQGVPI |
| 189.1 | Singleton | ACKPLLREDVTFQV | - | seq657 | ACKPLLREDVTFQV |
| 190.1 | Singleton | DSEPFPRQKHKKVD | - | seq820 | DSEPFPRQKHKKVD |
| 191.1 | Singleton | VGLRTPQRFTDLVKF | - | seq824 | VGLRTPQRFTDLVKF |
| 192.1 | Singleton | FCVQPEKGGRKPAR | - | seq886 | FCVQPEKGGRKPAR |
| 193.1 | Singleton | AHGDGRRPSKQRTFI | - | seq932 | AHGDGRRPSKQRTFI |
| 194.1 | Singleton | TWDDD | - | seq607 | TWDDD |
| 195.1 | Singleton | SPPEWLK | - | seq308 | SPPEWLK |
| 196.1 | Singleton | GSSYGF | - | seq768 | GSSYGF |
| 197.1 | Singleton | LSREM | - | seq1145 | LSREM |
| 198.1 | Singleton | LDPHAFHLLL | - | seq137 | LDPHAFHLLL |
| 199.1 | Singleton | HETHYGYATLSY | - | seq18 | HETHYGYATLSY |
| 200.1 | Singleton | TNLSVPNPLGFFPDH | - | seq641 | TNLSVPNPLGFFPDH |
| 201.1 | Singleton | SGLTSLF | - | seq1124 | SGLTSLF |
| 202.1 | Singleton | RIEDPPFNSLL | - | seq406 | RIEDPPFNSLL |
| 203.1 | Singleton | LDFELI | - | seq814 | LDFELI |
| 204.1 | Singleton | SASVPALTAVET | - | seq28 | SASVPALTAVET |
| 205.1 | Singleton | ETTTASGLVIPDTAK | - | seq124 | ETTTASGLVIPDTAK |
| 206.1 | Singleton | RTTSGLT | - | seq518 | RTTSGLT |
| 207.1 | Singleton | VETGHTSQVTPS | - | seq815 | VETGHTSQVTPS |
| 208.1 | Singleton | PEESDEDDFGMGGLF | - | seq280 | PEESDEDDFGMGGLF |
| 209.1 | Singleton | AFGVGDDESK | - | seq556 | AFGVGDDESK |
| 210.1 | Singleton | SYGFQY | - | seq334 | SYGFQY |
| 211.1 | Singleton | DQANPDYHYV | - | seq476 | DQANPDYHYV |
| 212.1 | Singleton | AIPLEVIK | - | seq407 | AIPLEVIK |
| 213.1 | Singleton | GSKVPEDT | - | seq532 | GSKVPEDT |
| 214.1 | Singleton | DKILVQAGEAETMTP | - | seq541 | DKILVQAGEAETMTP |
| 215.1 | Singleton | TESTVSSALAELATK | - | seq582 | TESTVSSALAELATK |
| 216.1 | Singleton | EATAESKSALTSVD | - | seq802 | EATAESKSALTSVD |
| 217.1 | Singleton | KPNRNGGGYYSASYS | - | seq29 | KPNRNGGGYYSASYS |
| 218.1 | Singleton | SRKAYDHNSP | - | seq597 | SRKAYDHNSP |
| 219.1 | Singleton | RVEFLV | - | seq1036 | RVEFLV |
| 220.1 | Singleton | QAQGLLTTVPAAPP | - | seq516 | QAQGLLTTVPAAPP |
| 221.1 | Singleton | SDKGATLTIKKEAFP | - | seq667 | SDKGATLTIKKEAFP |
| 222.1 | Singleton | RARGRGRGRGEKRP | - | seq32 | RARGRGRGRGEKRP |
| 223.1 | Singleton | NPKPQRKTKRNTNRR | - | seq827 | NPKPQRKTKRNTNRR |
| 224.1 | Singleton | KPNRPVVPSPDPN | - | seq38 | KPNRPVVPSPDPN |
| 225.1 | Singleton | EKDKPNRPVVPSP | - | seq143 | EKDKPNRPVVPSP |
| 226.1 | Singleton | VPSPDPNNSPARP | - | seq374 | VPSPDPNNSPARP |
| 227.1 | Singleton | QLRHLPSRVRYPHYS | - | seq80 | QLRHLPSRVRYPHYS |
| 228.1 | Singleton | TNSHVPILQERPPLE | - | seq285 | TNSHVPILQERPPLE |
| 229.1 | Singleton | DDSEPVLKGVKLHYT | - | seq323 | DDSEPVLKGVKLHYT |
| 230.1 | Singleton | PPPFNPRPSPYAELLK | - | seq429 | PPPFNPRPSPYAELLK |
| 231.1 | Singleton | HTSPDVDLGDISGIN | - | seq445 | HTSPDVDLGDISGIN |
| 232.1 | Singleton | RSPERCDLGDDLHLQ | - | seq461 | RSPERCDLGDDLHLQ |
| 233.1 | Singleton | NERTLDYHDSNVKNL | - | seq499 | NERTLDYHDSNVKNL |
| 234.1 | Singleton | PNASHPWDEDVMPDL | - | seq513 | PNASHPWDEDVMPDL |
| 235.1 | Singleton | EKNVTVTHSVNLLED | - | seq542 | EKNVTVTHSVNLLED |
| 236.1 | Singleton | PFNVNLKLQFLHDAF | - | seq544 | PFNVNLKLQFLHDAF |
| 237.1 | Singleton | NGIRRPKHLYVSMP | - | seq546 | NGIRRPKHLYVSMP |
| 238.1 | Singleton | TWNLDLNSLTTDQRLH | - | seq611 | TWNLDLNSLTTDQRLH |
| 239.1 | Singleton | SLSTNLDVTNSIEHQ | - | seq645 | SLSTNLDVTNSIEHQ |
| 240.1 | Singleton | LQTTGRPSHEAPN | - | seq672 | LQTTGRPSHEAPN |
| 241.1 | Singleton | VPNPLGFFPDHQLDP | - | seq729 | VPNPLGFFPDHQLDP |
| 242.1 | Singleton | DSPDADLIEANLLWR | - | seq749 | DSPDADLIEANLLWR |
| 243.1 | Singleton | SFQSFNLTEPHIT | - | seq758 | SFQSFNLTEPHIT |
| 244.1 | Singleton | KAGLLVSDGGPNLY | - | seq828 | KAGLLVSDGGPNLY |
| 245.1 | Singleton | PGLGAPVFHMTNYLE | - | seq894 | PGLGAPVFHMTNYLE |
| 246.1 | Singleton | QNPLAELKCSVKSFE | - | seq936 | QNPLAELKCSVKSFE |
| 247.1 | Singleton | SFKDILPKLTENPWQ | - | seq939 | SFKDILPKLTENPWQ |
| 248.1 | Singleton | LVEKPNLSSKRSELS | - | seq944 | LVEKPNLSSKRSELS |
| 249.1 | Singleton | NVFFLLLFSLTHFPL | - | seq982 | NVFFLLLFSLTHFPL |
| 250.1 | Singleton | CILRQLRHLPSRVRY | - | seq998 | CILRQLRHLPSRVRY |
| 251.1 | Singleton | SMTSPLLTWDGNKVT | - | seq1005 | SMTSPLLTWDGNKVT |
| 252.1 | Singleton | DPRYNQLLGPLPFRH | - | seq1031 | DPRYNQLLGPLPFRH |
| 253.1 | Singleton | KVLSPLQDNNDSGH | - | seq1057 | KVLSPLQDNNDSGH |
| 254.1 | Singleton | TQAHTTMRLANLFTSG | - | seq1085 | TQAHTTMRLANLFTSG |
| 255.1 | Singleton | NVTSIHSLLDEGKQSL | - | seq1094 | NVTSIHSLLDEGKQSL |
| 256.1 | Singleton | MANTGAMELV | - | seq989 | MANTGAMELV |
| 257.1 | Singleton | GGYYSASYSDPCSLK | - | seq42 | GGYYSASYSDPCSLK |
| 258.1 | Singleton | SSSDKPEA | - | seq415 | SSSDKPEA |
| 259.1 | Singleton | SSSAKPES | - | seq793 | SSSAKPES |
| 260.1 | Singleton | KPEASSSD | - | seq1033 | KPEASSSD |
| 261.1 | Singleton | YHSSVVHD | - | seq181 | YHSSVVHD |
| 262.1 | Singleton | SVRYDSPEFAGL | - | seq270 | SVRYDSPEFAGL |
| 263.1 | Singleton | GRHNSESYHAGF | - | seq590 | GRHNSESYHAGF |
| 264.1 | Singleton | ISNRDF | - | seq660 | ISNRDF |
| 265.1 | Singleton | MGFSYD | - | seq810 | MGFSYD |
| 266.1 | Singleton | VLSADFTP | - | seq929 | VLSADFTP |
| 267.1 | Singleton | SVWKDL | - | seq740 | SVWKDL |
| 268.1 | Singleton | PPSPEAHVPPPYVEP | - | seq47 | PPSPEAHVPPPYVEP |
| 269.1 | Singleton | LHEVDKDI | - | seq48 | LHEVDKDI |
| 270.1 | Singleton | EVDGDVKLSSNLVIL | - | seq166 | EVDGDVKLSSNLVIL |
| 271.1 | Singleton | PERIKLDYDQYCADV | - | seq216 | PERIKLDYDQYCADV |
| 272.1 | Singleton | DFVEEVLRKHPDKVE | - | seq298 | DFVEEVLRKHPDKVE |
| 273.1 | Singleton | EKRIENLNKKVDDGF | - | seq311 | EKRIENLNKKVDDGF |
| 274.1 | Singleton | WISEYKEVEILEADD | - | seq312 | WISEYKEVEILEADD |
| 275.1 | Singleton | VEFDLPGIKADSLDI | - | seq396 | VEFDLPGIKADSLDI |
| 276.1 | Singleton | LDEFKPIVQYDNFQD | - | seq414 | LDEFKPIVQYDNFQD |
| 277.1 | Singleton | CKWHVDNPIDEATA | - | seq501 | CKWHVDNPIDEATA |
| 278.1 | Singleton | QALDAHYDSVLKDI | - | seq510 | QALDAHYDSVLKDI |
| 279.1 | Singleton | DDEDLDEFKPIVQYD | - | seq547 | DDEDLDEFKPIVQYD |
| 280.1 | Singleton | LDVAEGDTVIYSKYG | - | seq581 | LDVAEGDTVIYSKYG |
| 281.1 | Singleton | NHVADIDKLIDYAA | - | seq674 | NHVADIDKLIDYAA |
| 282.1 | Singleton | EKVDNLGRSGGDIIK | - | seq691 | EKVDNLGRSGGDIIK |
| 283.1 | Singleton | DECHAVDSTTILGI | - | seq736 | DECHAVDSTTILGI |
| 284.1 | Singleton | LIYDAAVEGDLLFKL | - | seq978 | LIYDAAVEGDLLFKL |
| 285.1 | Singleton | LDKNHVADIDKLID | - | seq1091 | LDKNHVADIDKLID |
| 286.1 | Singleton | EDINGIRRPKHLYV | - | seq1144 | EDINGIRRPKHLYV |
| 287.1 | Singleton | GVVNQGPVDVPFSGKP | - | seq362 | GVVNQGPVDVPFSGKP |
| 288.1 | Singleton | PFSGKPLDERAVGGKG | - | seq399 | PFSGKPLDERAVGGKG |
| 289.1 | Singleton | QDPSKFTEPVKD | - | seq514 | QDPSKFTEPVKD |
| 290.1 | Singleton | ENYAFSMKNAVNDPN | - | seq51 | ENYAFSMKNAVNDPN |
| 291.1 | Singleton | NFNSNNYTYD | - | seq260 | NFNSNNYTYD |
| 292.1 | Singleton | YPVLVNFNSN | - | seq760 | YPVLVNFNSN |
| 293.1 | Singleton | LDFSLPSSSSYSY | - | seq112 | LDFSLPSSSSYSY |
| 294.1 | Singleton | PDSDAESYSSMPPL | - | seq128 | PDSDAESYSSMPPL |
| 295.1 | Singleton | LPQSPGPAFPLAE | - | seq174 | LPQSPGPAFPLAE |
| 296.1 | Singleton | SSMPPLEGEPGDPDL | - | seq203 | SSMPPLEGEPGDPDL |
| 297.1 | Singleton | YSELDDEEPMELDYP | - | seq218 | YSELDDEEPMELDYP |
| 298.1 | Singleton | KYFGGFNFSQILPDP | - | seq253 | KYFGGFNFSQILPDP |
| 299.1 | Singleton | VYYPDEIFRSDTLYL | - | seq255 | VYYPDEIFRSDTLYL |
| 300.1 | Singleton | ERVPLADVPLDDDS | - | seq265 | ERVPLADVPLDDDS |
| 301.1 | Singleton | GPGQEGLDQYGSIPL | - | seq284 | GPGQEGLDQYGSIPL |
| 302.1 | Singleton | LDDGYRPPPFNPRPSP | - | seq294 | LDDGYRPPPFNPRPSP |
| 303.1 | Singleton | TRLTSKGRPLVPT | - | seq314 | TRLTSKGRPLVPT |
| 304.1 | Singleton | QSGLGDNGYIPIPDE | - | seq342 | QSGLGDNGYIPIPDE |
| 305.1 | Singleton | PIPAASQLDLSGWF | - | seq427 | PIPAASQLDLSGWF |
| 306.1 | Singleton | SDTLYLTQDLFLPFY | - | seq441 | SDTLYLTQDLFLPFY |
| 307.1 | Singleton | KLFIRQEEVQQELYSP | - | seq529 | KLFIRQEEVQQELYSP |
| 308.1 | Singleton | YRLERPLLFALQCMP | - | seq530 | YRLERPLLFALQCMP |
| 309.1 | Singleton | KAMLFDGRSGEPFPY | - | seq612 | KAMLFDGRSGEPFPY |
| 310.1 | Singleton | LDPMVEERSDLEPS | - | seq666 | LDPMVEERSDLEPS |
| 311.1 | Singleton | DVESYSSMPPLEGEP | - | seq723 | DVESYSSMPPLEGEP |
| 312.1 | Singleton | ERSDLEPSIPSEYM | - | seq748 | ERSDLEPSIPSEYM |
| 313.1 | Singleton | LEDPASRDLVVSY | - | seq770 | LEDPASRDLVVSY |
| 314.1 | Singleton | VLVLFGFFTLSPWY | - | seq889 | VLVLFGFFTLSPWY |
| 315.1 | Singleton | PPLLTDDMIAAYTAA | - | seq896 | PPLLTDDMIAAYTAA |
| 316.1 | Singleton | GYQPYRVVVLSFELL | - | seq1013 | GYQPYRVVVLSFELL |
| 317.1 | Singleton | GYIPILGAPWLADLV | - | seq1015 | GYIPILGAPWLADLV |
| 318.1 | Singleton | FPSFAGLRPTFDTRL | - | seq1141 | FPSFAGLRPTFDTRL |
| 319.1 | Singleton | QIHRLVSGYDND | - | seq106 | QIHRLVSGYDND |
| 320.1 | Singleton | DTLCIGYHANNSTDT | - | seq387 | DTLCIGYHANNSTDT |
| 321.1 | Singleton | LYQDVNCTDVSTAIH | - | seq1117 | LYQDVNCTDVSTAIH |
| 322.1 | Singleton | TPPMPSIGLEEEE | - | seq62 | TPPMPSIGLEEEE |
| 323.1 | Singleton | SDKPDNKP | - | seq880 | SDKPDNKP |
| 324.1 | Singleton | EDVEKDKPNRPVV | - | seq67 | EDVEKDKPNRPVV |
| 325.1 | Singleton | PLAEDVEKDKPNR | - | seq148 | PLAEDVEKDKPNR |
| 326.1 | Singleton | MPSIGLEEEEEEE | - | seq69 | MPSIGLEEEEEEE |
| 327.1 | Singleton | EEEEGAGDGEHLE | - | seq70 | EEEEGAGDGEHLE |
| 328.1 | Singleton | IGLEEEEEEEGAG | - | seq244 | IGLEEEEEEEGAG |
| 329.1 | Singleton | EEEEEEEGAGDGE | - | seq472 | EEEEEEEGAGDGE |
| 330.1 | Singleton | LGDDTGIHVI | - | seq288 | LGDDTGIHVI |
| 331.1 | Singleton | DSYIII | - | seq613 | DSYIII |
| 332.1 | Singleton | PAEAGKTELDDGYRPP | - | seq141 | PAEAGKTELDDGYRPP |
| 333.1 | Singleton | EGGDGTRDTLPQS | - | seq208 | EGGDGTRDTLPQS |
| 334.1 | Singleton | DGTRDTLPQSPGP | - | seq267 | DGTRDTLPQSPGP |
| 335.1 | Singleton | GEPGDPDLSDGSWST | - | seq608 | GEPGDPDLSDGSWST |
| 336.1 | Singleton | WGPSSDPAWERNDPT | - | seq757 | WGPSSDPAWERNDPT |
| 337.1 | Singleton | PAGRNNDGGSSAPTPK | - | seq934 | PAGRNNDGGSSAPTPK |
| 338.1 | Singleton | DSIYYVDANASIQEM | - | seq454 | DSIYYVDANASIQEM |
| 339.1 | Singleton | WHVLYSP | - | seq154 | WHVLYSP |
| 340.1 | Singleton | PSSRRYPFV | - | seq791 | PSSRRYPFV |
| 341.1 | Singleton | EGAGDGEHLEGGD | - | seq81 | EGAGDGEHLEGGD |
| 342.1 | Singleton | GDGEHLEGGDGTR | - | seq102 | GDGEHLEGGDGTR |
| 343.1 | Singleton | PAPSGCPPDSDAES | - | seq82 | PAPSGCPPDSDAES |
| 344.1 | Singleton | RDTLPQSPGPAFP | - | seq219 | RDTLPQSPGPAFP |
| 345.1 | Singleton | RYSAPPGDPPQPEYD | - | seq269 | RYSAPPGDPPQPEYD |
| 346.1 | Singleton | QEPSGDGGGNDAGNN | - | seq632 | QEPSGDGGGNDAGNN |
| 347.1 | Singleton | PTRAPSGPRPP | - | seq871 | PTRAPSGPRPP |
| 348.1 | Singleton | MSAAYDNIYT | - | seq617 | MSAAYDNIYT |
| 349.1 | Singleton | ETWKRPDYEPPTVLG | - | seq378 | ETWKRPDYEPPTVLG |
| 350.1 | Singleton | PEARLISVRYDS | - | seq595 | PEARLISVRYDS |
| 351.1 | Singleton | REEVSFRVGLHEYP | - | seq787 | REEVSFRVGLHEYP |
| 352.1 | Singleton | RAVVAPDKEVLYE | - | seq1000 | RAVVAPDKEVLYE |
| 353.1 | Singleton | SGPEGSGGSGPQRRGGD | - | seq110 | SGPEGSGGSGPQRRGGD |
| 354.1 | Singleton | ASSGIGPE | - | seq923 | ASSGIGPE |
| 355.1 | Singleton | SPGPAFPLAEDVE | - | seq275 | SPGPAFPLAEDVE |
| 356.1 | Singleton | DTAWDF | - | seq394 | DTAWDF |
| 357.1 | Singleton | PAFPLAEDVEKDK | - | seq157 | PAFPLAEDVEKDK |
| 358.1 | Singleton | FEPLRAETDDVEPS | - | seq551 | FEPLRAETDDVEPS |
| 359.1 | Singleton | YGYATLSYADYW | - | seq96 | YGYATLSYADYW |
| 360.1 | Singleton | DCHAPTYLPAEVDGD | - | seq100 | DCHAPTYLPAEVDGD |
| 361.1 | Singleton | EHLEGGDGTRDTL | - | seq133 | EHLEGGDGTRDTL |
| 362.1 | Singleton | TSKGRPLVPTPQH | - | seq104 | TSKGRPLVPTPQH |
| 363.1 | Singleton | DIDLGESRVVIQDYD | - | seq105 | DIDLGESRVVIQDYD |
| 364.1 | Singleton | LDQVLDYV | - | seq762 | LDQVLDYV |
| 365.1 | Singleton | QIVGGVYL | - | seq1123 | QIVGGVYL |
| 366.1 | Singleton | RISNLND | - | seq599 | RISNLND |
| 367.1 | Singleton | QDADYHRV | - | seq701 | QDADYHRV |
| 368.1 | Singleton | VPDVDSRGAILRRQY | - | seq987 | VPDVDSRGAILRRQY |
| 369.1 | Singleton | LKLYFEP | - | seq192 | LKLYFEP |
| 370.1 | Singleton | NPPLVETWKKPDYEPP | - | seq385 | NPPLVETWKKPDYEPP |
| 371.1 | Singleton | DPVGHPAAPRAPGPE | - | seq388 | DPVGHPAAPRAPGPE |
| 372.1 | Singleton | RPSFSFFAVGPDGMP | - | seq891 | RPSFSFFAVGPDGMP |
| 373.1 | Singleton | SIYKMNNEIVNSTID | - | seq353 | SIYKMNNEIVNSTID |
| 374.1 | Singleton | AFFSILQDMRNTIM | - | seq498 | AFFSILQDMRNTIM |
| 375.1 | Singleton | GFSEIMRSTLEKDNT | - | seq742 | GFSEIMRSTLEKDNT |
| 376.1 | Singleton | LTDIQEDITRHEQQ | - | seq836 | LTDIQEDITRHEQQ |
| 377.1 | Singleton | SESTIGDVLQQMAI | - | seq913 | SESTIGDVLQQMAI |
| 378.1 | Singleton | ARGACVTIMTVDNPA | - | seq1097 | ARGACVTIMTVDNPA |
| 379.1 | Singleton | TGNMISNQAKYVSDT | - | seq1100 | TGNMISNQAKYVSDT |
| 380.1 | Singleton | TQNISGTQVYQDPAI | - | seq1101 | TQNISGTQVYQDPAI |
| 381.1 | Singleton | VAAEEYVEVTRVGDF | - | seq114 | VAAEEYVEVTRVGDF |
| 382.1 | Singleton | EYVEVTRVGDFHYVT | - | seq186 | EYVEVTRVGDFHYVT |
| 383.1 | Singleton | GDVEEAIERAVV | - | seq584 | GDVEEAIERAVV |
| 384.1 | Singleton | EEASVTVVEGQVDYY | - | seq700 | EEASVTVVEGQVDYY |
| 385.1 | Singleton | RYGNVLDVNAIDIE | - | seq781 | RYGNVLDVNAIDIE |
| 386.1 | Singleton | KKAEEAKKIVDKI | - | seq119 | KKAEEAKKIVDKI |
| 387.1 | Singleton | GDREIGEK | - | seq405 | GDREIGEK |
| 388.1 | Singleton | GIGPEKAF | - | seq938 | GIGPEKAF |
| 389.1 | Singleton | MRFDRGYI | - | seq984 | MRFDRGYI |
| 390.1 | Singleton | PDDDPQPGPSREYRY | - | seq123 | PDDDPQPGPSREYRY |
| 391.1 | Singleton | EGQDRGYSY | - | seq1061 | EGQDRGYSY |
| 392.1 | Singleton | MAILGD | - | seq483 | MAILGD |
| 393.1 | Singleton | DGYPIINYEYAIVNN | - | seq520 | DGYPIINYEYAIVNN |
| 394.1 | Singleton | YVYKGYQPIDVVRDL | - | seq860 | YVYKGYQPIDVVRDL |
| 395.1 | Singleton | AKKAILITDAAKDKG | - | seq127 | AKKAILITDAAKDKG |
| 396.1 | Singleton | GADLAGADLA | - | seq848 | GADLAGADLA |
| 397.1 | Singleton | SEYPSQPSS | - | seq1088 | SEYPSQPSS |
| 398.1 | Singleton | FKSDPKKSDVKTYF | - | seq129 | FKSDPKKSDVKTYF |
| 399.1 | Singleton | QSSWPKLEQ | - | seq132 | QSSWPKLEQ |
| 400.1 | Singleton | VDTVGTGYHSRF | - | seq134 | VDTVGTGYHSRF |
| 401.1 | Singleton | YCFTPSPVVVGTTDR | - | seq356 | YCFTPSPVVVGTTDR |
| 402.1 | Singleton | AICKRIPDKKPGKKT | - | seq135 | AICKRIPDKKPGKKT |
| 403.1 | Singleton | VLVVLLLFAGVDAETH | - | seq832 | VLVVLLLFAGVDAETH |
| 404.1 | Singleton | TLVNADANYL | - | seq908 | TLVNADANYL |
| 405.1 | Singleton | DGKPCTPPALNCYWP | - | seq985 | DGKPCTPPALNCYWP |
| 406.1 | Singleton | RVPRIPRDPRPPRPP | - | seq775 | RVPRIPRDPRPPRPP |
| 407.1 | Singleton | GPPYRYEPEKFT | - | seq222 | GPPYRYEPEKFT |
| 408.1 | Singleton | EAELTGYG | - | seq779 | EAELTGYG |
| 409.1 | Singleton | AEAEGGTW | - | seq858 | AEAEGGTW |
| 410.1 | Singleton | RGQKAKSNPNRPLPV | - | seq436 | RGQKAKSNPNRPLPV |
| 411.1 | Singleton | VKEVASKTND | - | seq625 | VKEVASKTND |
| 412.1 | Singleton | YIGVAPRKAIPA | - | seq644 | YIGVAPRKAIPA |
| 413.1 | Singleton | TPKDHIGTRNPNNNA | - | seq892 | TPKDHIGTRNPNNNA |
| 414.1 | Singleton | KLRDVKDNKSTDVKL | - | seq145 | KLRDVKDNKSTDVKL |
| 415.1 | Singleton | SIKEDVQF | - | seq481 | SIKEDVQF |
| 416.1 | Singleton | KQNEPEDI | - | seq799 | KQNEPEDI |
| 417.1 | Singleton | PGFGDRRKAMLEDIA | - | seq302 | PGFGDRRKAMLEDIA |
| 418.1 | Singleton | WDEDGAKRIPVDVSE | - | seq462 | WDEDGAKRIPVDVSE |
| 419.1 | Singleton | EIPFYGKAIPIETIKG | - | seq572 | EIPFYGKAIPIETIKG |
| 420.1 | Singleton | LCENPEWAPLKDNRI | - | seq893 | LCENPEWAPLKDNRI |
| 421.1 | Singleton | FRGKELKKSIQPDEA | - | seq950 | FRGKELKKSIQPDEA |
| 422.1 | Singleton | EIDGKQTHQSVAISR | - | seq969 | EIDGKQTHQSVAISR |
| 423.1 | Singleton | YEQLNDSSEEEDEIDG | - | seq149 | YEQLNDSSEEEDEIDG |
| 424.1 | Singleton | FCGRIHTRYSSAYEL | - | seq185 | FCGRIHTRYSSAYEL |
| 425.1 | Singleton | REPLENALTVFTD | - | seq303 | REPLENALTVFTD |
| 426.1 | Singleton | MLMRTDPFRELDRFA | - | seq321 | MLMRTDPFRELDRFA |
| 427.1 | Singleton | LGDVGEAFVDSLTSQ | - | seq370 | LGDVGEAFVDSLTSQ |
| 428.1 | Singleton | VTHESYQELVKKLEA | - | seq393 | VTHESYQELVKKLEA |
| 429.1 | Singleton | TCELFSRVEDVLGLP | - | seq430 | TCELFSRVEDVLGLP |
| 430.1 | Singleton | LREQLSSVSSFERFE | - | seq474 | LREQLSSVSSFERFE |
| 431.1 | Singleton | KFLNPDREYDFRDLT | - | seq480 | KFLNPDREYDFRDLT |
| 432.1 | Singleton | ALTGLGDKFGESIVN | - | seq527 | ALTGLGDKFGESIVN |
| 433.1 | Singleton | SVSRARTRHLLLCL | - | seq548 | SVSRARTRHLLLCL |
| 434.1 | Singleton | CNSRQTDREDELI | - | seq578 | CNSRQTDREDELI |
| 435.1 | Singleton | HTFDDFCPECRPLGL | - | seq615 | HTFDDFCPECRPLGL |
| 436.1 | Singleton | ENSANPPPPDRSLPTP | - | seq662 | ENSANPPPPDRSLPTP |
| 437.1 | Singleton | RSELSQLSMYRVFEV | - | seq690 | RSELSQLSMYRVFEV |
| 438.1 | Singleton | SEATGLSSEKITKL | - | seq710 | SEATGLSSEKITKL |
| 439.1 | Singleton | RLITGRLQSLQTYVT | - | seq741 | RLITGRLQSLQTYVT |
| 440.1 | Singleton | RGLGEAQLGNSSGNF | - | seq744 | RGLGEAQLGNSSGNF |
| 441.1 | Singleton | ILFCCSKEKRKKKQAAT | - | seq766 | ILFCCSKEKRKKKQAAT |
| 442.1 | Singleton | GELDNRGSQFYLAMY | - | seq769 | GELDNRGSQFYLAMY |
| 443.1 | Singleton | WMLRAAEEYSMNLGF | - | seq804 | WMLRAAEEYSMNLGF |
| 444.1 | Singleton | DSTLRLCVQSTHVDI | - | seq839 | DSTLRLCVQSTHVDI |
| 445.1 | Singleton | QLVLGENLDTERILAS | - | seq843 | QLVLGENLDTERILAS |
| 446.1 | Singleton | RLCVQSTHVDIRTLE | - | seq844 | RLCVQSTHVDIRTLE |
| 447.1 | Singleton | DWTKVTLDGRPLSTIQ | - | seq854 | DWTKVTLDGRPLSTIQ |
| 448.1 | Singleton | TLTSGSDLDRCTTFD | - | seq878 | TLTSGSDLDRCTTFD |
| 449.1 | Singleton | TIRGQFSNMSLSLLD | - | seq902 | TIRGQFSNMSLSLLD |
| 450.1 | Singleton | QYVLATYDTSRVEHA | - | seq903 | QYVLATYDTSRVEHA |
| 451.1 | Singleton | RPGLDELSFTLT | - | seq904 | RPGLDELSFTLT |
| 452.1 | Singleton | FRMELLSLPQDEWAG | - | seq912 | FRMELLSLPQDEWAG |
| 453.1 | Singleton | VETLDIVTRSPTFS | - | seq941 | VETLDIVTRSPTFS |
| 454.1 | Singleton | FGLQLELTEGMRFDK | - | seq1019 | FGLQLELTEGMRFDK |
| 455.1 | Singleton | WRSLDVEMTAVQRSF | - | seq1024 | WRSLDVEMTAVQRSF |
| 456.1 | Singleton | NGEEYLILSARDVLA | - | seq1047 | NGEEYLILSARDVLA |
| 457.1 | Singleton | QEAGNFERISGDLKT | - | seq1071 | QEAGNFERISGDLKT |
| 458.1 | Singleton | LGVRATRKTSERSQP | - | seq1073 | LGVRATRKTSERSQP |
| 459.1 | Singleton | NKNLQEEISDLTEQL | - | seq1084 | NKNLQEEISDLTEQL |
| 460.1 | Singleton | MTLLELSDFVKKFEE | - | seq1120 | MTLLELSDFVKKFEE |
| 461.1 | Singleton | ILTGGKSKQTEGLLLL | - | seq1127 | ILTGGKSKQTEGLLLL |
| 462.1 | Singleton | GKDSADKMPLETQLA | - | seq1130 | GKDSADKMPLETQLA |
| 463.1 | Singleton | QFAPSASAFFGMSRI | - | seq152 | QFAPSASAFFGMSRI |
| 464.1 | Singleton | AGTVNIGASDAYLSE | - | seq488 | AGTVNIGASDAYLSE |
| 465.1 | Singleton | DNSGDQRQVDLTPKK | - | seq167 | DNSGDQRQVDLTPKK |
| 466.1 | Singleton | FVDSIGQSFQNTWEG | - | seq183 | FVDSIGQSFQNTWEG |
| 467.1 | Singleton | INPWDSKSDYLG | - | seq187 | INPWDSKSDYLG |
| 468.1 | Singleton | NWTHCFDPQIQAIVS | - | seq206 | NWTHCFDPQIQAIVS |
| 469.1 | Singleton | LPRSNTSSGAIPPAD | - | seq227 | LPRSNTSSGAIPPAD |
| 470.1 | Singleton | DNKSTDVKLPVVKAE | - | seq232 | DNKSTDVKLPVVKAE |
| 471.1 | Singleton | TSVDQVLDEISEAT | - | seq305 | TSVDQVLDEISEAT |
| 472.1 | Singleton | ITSAGRHPDSDIFLD | - | seq309 | ITSAGRHPDSDIFLD |
| 473.1 | Singleton | LNDITKEYEKLLNEI | - | seq327 | LNDITKEYEKLLNEI |
| 474.1 | Singleton | NSTDTVDTVLEKNVT | - | seq351 | NSTDTVDTVLEKNVT |
| 475.1 | Singleton | CTIAALGSSDRDTVV | - | seq354 | CTIAALGSSDRDTVV |
| 476.1 | Singleton | SKVEQEETNPEVLIK | - | seq383 | SKVEQEETNPEVLIK |
| 477.1 | Singleton | TKKNYSELDDEEPME | - | seq403 | TKKNYSELDDEEPME |
| 478.1 | Singleton | EQNKKFFADKPDESTL | - | seq410 | EQNKKFFADKPDESTL |
| 479.1 | Singleton | NVLDVNAIDIEEPS | - | seq420 | NVLDVNAIDIEEPS |
| 480.1 | Singleton | SLGADSSIAYSNNTI | - | seq470 | SLGADSSIAYSNNTI |
| 481.1 | Singleton | ITYNESHSSDQRPY | - | seq485 | ITYNESHSSDQRPY |
| 482.1 | Singleton | IFLLFLTLTSGSDLD | - | seq507 | IFLLFLTLTSGSDLD |
| 483.1 | Singleton | PVSMAKTSVDCNMYI | - | seq525 | PVSMAKTSVDCNMYI |
| 484.1 | Singleton | INLRTNNSATIV | - | seq554 | INLRTNNSATIV |
| 485.1 | Singleton | IMSGEVPSTEDLVN | - | seq629 | IMSGEVPSTEDLVN |
| 486.1 | Singleton | TVKLYTSVENAQQDK | - | seq735 | TVKLYTSVENAQQDK |
| 487.1 | Singleton | TSEILDISPCAFGGV | - | seq786 | TSEILDISPCAFGGV |
| 488.1 | Singleton | KVISELNGKNIEDVI | - | seq806 | KVISELNGKNIEDVI |
| 489.1 | Singleton | NGTYDYPKYSEEAKL | - | seq807 | NGTYDYPKYSEEAKL |
| 490.1 | Singleton | ISNCVADYSVLYNST | - | seq835 | ISNCVADYSVLYNST |
| 491.1 | Singleton | GNFGDQDLIRQGTDY | - | seq841 | GNFGDQDLIRQGTDY |
| 492.1 | Singleton | VDDGFLDIWTYNAEL | - | seq890 | VDDGFLDIWTYNAEL |
| 493.1 | Singleton | DLGDISGINASVVNI | - | seq898 | DLGDISGINASVVNI |
| 494.1 | Singleton | VNNTDTNFHSDITFR | - | seq910 | VNNTDTNFHSDITFR |
| 495.1 | Singleton | AWDMMMNWSPTTALV | - | seq920 | AWDMMMNWSPTTALV |
| 496.1 | Singleton | IEEVALSNTGEIPF | - | seq951 | IEEVALSNTGEIPF |
| 497.1 | Singleton | YTSILYGNGPGYA | - | seq953 | YTSILYGNGPGYA |
| 498.1 | Singleton | EFGLDGVTYEIDLTN | - | seq961 | EFGLDGVTYEIDLTN |
| 499.1 | Singleton | TDDRINASDWPSMKS | - | seq967 | TDDRINASDWPSMKS |
| 500.1 | Singleton | GNPNRPDGGILT | - | seq972 | GNPNRPDGGILT |
| 501.1 | Singleton | FISCNNSGKDGNTSA | - | seq1039 | FISCNNSGKDGNTSA |
| 502.1 | Singleton | APRITFGGPTDSTDN | - | seq1065 | APRITFGGPTDSTDN |
| 503.1 | Singleton | SVDCNMYICGDSTEC | - | seq1069 | SVDCNMYICGDSTEC |
| 504.1 | Singleton | NAFLTALTNAGIAYD | - | seq1077 | NAFLTALTNAGIAYD |
| 505.1 | Singleton | LGWKVSDLKSSTAVI | - | seq1087 | LGWKVSDLKSSTAVI |
| 506.1 | Singleton | PSKALDSKSYTSI | - | seq1106 | PSKALDSKSYTSI |
| 507.1 | Singleton | DWVCSILTDFKNWL | - | seq1109 | DWVCSILTDFKNWL |
| 508.1 | Singleton | SANNDAEIGNLI | - | seq1128 | SANNDAEIGNLI |
| 509.1 | Singleton | RAILTAFSPAQDIWG | - | seq1131 | RAILTAFSPAQDIWG |
| 510.1 | Singleton | HYTNESKFKFILE | - | seq1137 | HYTNESKFKFILE |
| 511.1 | Singleton | NTRNIDATSTGN | - | seq1142 | NTRNIDATSTGN |
| 512.1 | Singleton | SSSGCVTG | - | seq158 | SSSGCVTG |
| 513.1 | Singleton | AGSGIIISDTPVHDC | - | seq202 | AGSGIIISDTPVHDC |
| 514.1 | Singleton | GSAKSAAGTASHVSI | - | seq1080 | GSAKSAAGTASHVSI |
| 515.1 | Singleton | LMDAAVDVFADG | - | seq482 | LMDAAVDVFADG |
| 516.1 | Singleton | VAESDGVDEDRAAL | - | seq619 | VAESDGVDEDRAAL |
| 517.1 | Singleton | DDLVGAGVIDAVA | - | seq1052 | DDLVGAGVIDAVA |
| 518.1 | Singleton | AVLLGACS | - | seq1146 | AVLLGACS |
| 519.1 | Singleton | QNVLSADF | - | seq1072 | QNVLSADF |
| 520.1 | Singleton | GFYVLENDYS | - | seq494 | GFYVLENDYS |
| 521.1 | Singleton | PAADRAAAPDAVAA | - | seq165 | PAADRAAAPDAVAA |
| 522.1 | Singleton | VSGGSWVDIVLE | - | seq638 | VSGGSWVDIVLE |
| 523.1 | Singleton | LLTTNTTGNPEY | - | seq745 | LLTTNTTGNPEY |
| 524.1 | Singleton | DTLNGGEYTVFAPTN | - | seq840 | DTLNGGEYTVFAPTN |
| 525.1 | Singleton | EKPTINTTRTNI | - | seq977 | EKPTINTTRTNI |
| 526.1 | Singleton | QELPPPNAQEL | - | seq171 | QELPPPNAQEL |
| 527.1 | Singleton | KFAELLEQQKNAQFP | - | seq496 | KFAELLEQQKNAQFP |
| 528.1 | Singleton | YRDVVADVRFLP | - | seq240 | YRDVVADVRFLP |
| 529.1 | Singleton | FDNEDQRV | - | seq825 | FDNEDQRV |
| 530.1 | Singleton | INRIADQAQY | - | seq885 | INRIADQAQY |
| 531.1 | Singleton | HVDTSYECDIPIGAG | - | seq313 | HVDTSYECDIPIGAG |
| 532.1 | Singleton | FQTQAGCLIGAEHVD | - | seq800 | FQTQAGCLIGAEHVD |
| 533.1 | Singleton | VRNVSGVYHVTNDCS | - | seq834 | VRNVSGVYHVTNDCS |
| 534.1 | Singleton | ITDHEEDN | - | seq528 | ITDHEEDN |
| 535.1 | Singleton | FLVNTW | - | seq1021 | FLVNTW |
| 536.1 | Singleton | STVSEEASAEDVVC | - | seq184 | STVSEEASAEDVVC |
| 537.1 | Singleton | RGYISQYF | - | seq968 | RGYISQYF |
| 538.1 | Singleton | GIFRAAVCTR | - | seq1014 | GIFRAAVCTR |
| 539.1 | Singleton | SSSDKPDN | - | seq335 | SSSDKPDN |
| 540.1 | Singleton | SRSESSIENF | - | seq189 | SRSESSIENF |
| 541.1 | Singleton | YPGDFIDYEELREQL | - | seq381 | YPGDFIDYEELREQL |
| 542.1 | Singleton | YTFKEDEYPSTAYLQ | - | seq549 | YTFKEDEYPSTAYLQ |
| 543.1 | Singleton | HLREFVFKNKDGFLY | - | seq976 | HLREFVFKNKDGFLY |
| 544.1 | Singleton | EGWGKSPGFGTTVDF | - | seq1113 | EGWGKSPGFGTTVDF |
| 545.1 | Singleton | CPPRRRAG | - | seq1050 | CPPRRRAG |
| 546.1 | Singleton | PQTWWEKNAKDIWEG | - | seq200 | PQTWWEKNAKDIWEG |
| 547.1 | Singleton | GACSCGSCCKFDEDD | - | seq201 | GACSCGSCCKFDEDD |
| 548.1 | Singleton | GDEFDTYDDIKKVT | - | seq231 | GDEFDTYDDIKKVT |
| 549.1 | Singleton | GTRIRFKDDTSFED | - | seq478 | GTRIRFKDDTSFED |
| 550.1 | Singleton | SAMSTTDLEAYFKDC | - | seq575 | SAMSTTDLEAYFKDC |
| 551.1 | Singleton | EYETDPSVTKMV | - | seq943 | EYETDPSVTKMV |
| 552.1 | Singleton | RADESKTDPQTEA | - | seq956 | RADESKTDPQTEA |
| 553.1 | Singleton | GTKSLEPCTCGAVD | - | seq207 | GTKSLEPCTCGAVD |
| 554.1 | Singleton | RGPEQTQGNFGDQDL | - | seq366 | RGPEQTQGNFGDQDL |
| 555.1 | Singleton | PAFGANSNNPDWDFNP | - | seq209 | PAFGANSNNPDWDFNP |
| 556.1 | Singleton | VVIRACNFELCDNPF | - | seq213 | VVIRACNFELCDNPF |
| 557.1 | Singleton | DKEVLYQQY | - | seq862 | DKEVLYQQY |
| 558.1 | Singleton | CYWPLNDYGFYTTTG | - | seq655 | CYWPLNDYGFYTTTG |
| 559.1 | Singleton | LIEEGQRMA | - | seq914 | LIEEGQRMA |
| 560.1 | Singleton | VFGSTMNNKSQSVII | - | seq948 | VFGSTMNNKSQSVII |
| 561.1 | Singleton | DLYCYEQLNDSSEE | - | seq225 | DLYCYEQLNDSSEE |
| 562.1 | Singleton | VYDFAFQDL | - | seq750 | VYDFAFQDL |
| 563.1 | Singleton | QKNACSFDLCNSYDVL | - | seq1096 | QKNACSFDLCNSYDVL |
| 564.1 | Singleton | YEEQMQDAFETGVMF | - | seq229 | YEEQMQDAFETGVMF |
| 565.1 | Singleton | FDARTMQY | - | seq1076 | FDARTMQY |
| 566.1 | Singleton | YPWPLYGNEG | - | seq230 | YPWPLYGNEG |
| 567.1 | Singleton | PAGGAYSMYTNWEQD | - | seq755 | PAGGAYSMYTNWEQD |
| 568.1 | Singleton | GYPVHKPVTAGWNGY | - | seq966 | GYPVHKPVTAGWNGY |
| 569.1 | Singleton | LVTKDIDFD | - | seq651 | LVTKDIDFD |
| 570.1 | Singleton | AVEVDPDDVNKNTL | - | seq252 | AVEVDPDDVNKNTL |
| 571.1 | Singleton | MESPHDLVVDTV | - | seq263 | MESPHDLVVDTV |
| 572.1 | Singleton | VVSWKNKEL | - | seq1112 | VVSWKNKEL |
| 573.1 | Singleton | REKKLADRAFLDQK | - | seq517 | REKKLADRAFLDQK |
| 574.1 | Singleton | RQKLKDAERAVEVD | - | seq822 | RQKLKDAERAVEVD |
| 575.1 | Singleton | ICESAGVQEDAASL | - | seq805 | ICESAGVQEDAASL |
| 576.1 | Singleton | DERQQEPEEPVSQRAS | - | seq278 | DERQQEPEEPVSQRAS |
| 577.1 | Singleton | TEVEDDREPSVPSEY | - | seq345 | TEVEDDREPSVPSEY |
| 578.1 | Singleton | SRQPALEQEVPES | - | seq777 | SRQPALEQEVPES |
| 579.1 | Singleton | QASDDGDKGSDVES | - | seq246 | QASDDGDKGSDVES |
| 580.1 | Singleton | DDGDKGSDVESYSSM | - | seq553 | DDGDKGSDVESYSSM |
| 581.1 | Singleton | TTHGKAYDVDMVDA | - | seq247 | TTHGKAYDVDMVDA |
| 582.1 | Singleton | TTHKTAYDCDMVDA | - | seq274 | TTHKTAYDCDMVDA |
| 583.1 | Singleton | EEVIDTMKSMQRDED | - | seq307 | EEVIDTMKSMQRDED |
| 584.1 | Singleton | SVLCPRRGHKKTV | - | seq550 | SVLCPRRGHKKTV |
| 585.1 | Singleton | VPPPRKKRTVVLTE | - | seq680 | VPPPRKKRTVVLTE |
| 586.1 | Singleton | ESKQLTVKQKTEQIK | - | seq796 | ESKQLTVKQKTEQIK |
| 587.1 | Singleton | FSEVEGRIQDLEKYV | - | seq861 | FSEVEGRIQDLEKYV |
| 588.1 | Singleton | AKVPSKRTQKRHRLI | - | seq870 | AKVPSKRTQKRHRLI |
| 589.1 | Singleton | KKTDEAQPLPQRQKK | - | seq1046 | KKTDEAQPLPQRQKK |
| 590.1 | Singleton | GYLPISGEMEKVT | - | seq1119 | GYLPISGEMEKVT |
| 591.1 | Singleton | ITKLTPEELENLAK | - | seq434 | ITKLTPEELENLAK |
| 592.1 | Singleton | NTIMASKSVGTAE | - | seq765 | NTIMASKSVGTAE |
| 593.1 | Singleton | GCPLPSTKAPPIPPP | - | seq900 | GCPLPSTKAPPIPPP |
| 594.1 | Singleton | SQLKNNAKEIGNGCF | - | seq971 | SQLKNNAKEIGNGCF |
| 595.1 | Singleton | GVEVTLAEKCEKEFGI | - | seq990 | GVEVTLAEKCEKEFGI |
| 596.1 | Singleton | TYRATYQD | - | seq816 | TYRATYQD |
| 597.1 | Singleton | VDGVHAPDKEVKVHA | - | seq256 | VDGVHAPDKEVKVHA |
| 598.1 | Singleton | GAANPSDDSSDSDAK | - | seq594 | GAANPSDDSSDSDAK |
| 599.1 | Singleton | YSSVQEPQAKVPSKR | - | seq863 | YSSVQEPQAKVPSKR |
| 600.1 | Singleton | KEAPTTKPTEKP | - | seq258 | KEAPTTKPTEKP |
| 601.1 | Singleton | RDEVDFCVGLNSFV | - | seq330 | RDEVDFCVGLNSFV |
| 602.1 | Singleton | AFLPVGAFNSDNFKS | - | seq452 | AFLPVGAFNSDNFKS |
| 603.1 | Singleton | HLGGEDFDNRLVAHF | - | seq817 | HLGGEDFDNRLVAHF |
| 604.1 | Singleton | HTSSMRGVYYPDEIF | - | seq332 | HTSSMRGVYYPDEIF |
| 605.1 | Singleton | PEFAGLSGSVQY | - | seq677 | PEFAGLSGSVQY |
| 606.1 | Singleton | EDDDDDFGMGALF | - | seq272 | EDDDDDFGMGALF |
| 607.1 | Singleton | GEPQSSIIQY | - | seq618 | GEPQSSIIQY |
| 608.1 | Singleton | RQAQDIQPV | - | seq1027 | RQAQDIQPV |
| 609.1 | Singleton | QGTDYKHW | - | seq1133 | QGTDYKHW |
| 610.1 | Singleton | RGLAQALIDQ | - | seq795 | RGLAQALIDQ |
| 611.1 | Singleton | PKPKVASEAFMST | - | seq831 | PKPKVASEAFMST |
| 612.1 | Singleton | RRREGPDRSPR | - | seq803 | RRREGPDRSPR |
| 613.1 | Singleton | RRPEGRTW | - | seq1105 | RRPEGRTW |
| 614.1 | Singleton | VSTRGRIPADVIDA | - | seq369 | VSTRGRIPADVIDA |
| 615.1 | Singleton | HTINHTFGNPVIPFK | - | seq376 | HTINHTFGNPVIPFK |
| 616.1 | Singleton | STGADTGGDIVQQP | - | seq1058 | STGADTGGDIVQQP |
| 617.1 | Singleton | PDVQRRVQQEIDDVI | - | seq300 | PDVQRRVQQEIDDVI |
| 618.1 | Singleton | WLTYHGAIKLDDKDP | - | seq301 | WLTYHGAIKLDDKDP |
| 619.1 | Singleton | EKMITEFE | - | seq675 | EKMITEFE |
| 620.1 | Singleton | KPSPWAPKKHRRLS | - | seq583 | KPSPWAPKKHRRLS |
| 621.1 | Singleton | SALEWIDLDSSL | - | seq310 | SALEWIDLDSSL |
| 622.1 | Singleton | MYASSGIG | - | seq1054 | MYASSGIG |
| 623.1 | Singleton | QPQPQPQPQPQPQQF | - | seq322 | QPQPQPQPQPQPQQF |
| 624.1 | Singleton | TKKFDEVVKANGGYL | - | seq945 | TKKFDEVVKANGGYL |
| 625.1 | Singleton | WQETVKLGCYVEA | - | seq1143 | WQETVKLGCYVEA |
| 626.1 | Singleton | SNTVKVVGKGGHS | - | seq328 | SNTVKVVGKGGHS |
| 627.1 | Singleton | VGDLCGSVFL | - | seq503 | VGDLCGSVFL |
| 628.1 | Singleton | VFGGAASCAAPIQAD | - | seq921 | VFGGAASCAAPIQAD |
| 629.1 | Singleton | WSNGQPVTAADFVYA | - | seq382 | WSNGQPVTAADFVYA |
| 630.1 | Singleton | NLNFEMYGAVYSVN | - | seq467 | NLNFEMYGAVYSVN |
| 631.1 | Singleton | TRKSIRIGPGQAFY | - | seq678 | TRKSIRIGPGQAFY |
| 632.1 | Singleton | FDRFKGSGPGYY | - | seq705 | FDRFKGSGPGYY |
| 633.1 | Singleton | APNYTQHTSSMRGVY | - | seq759 | APNYTQHTSSMRGVY |
| 634.1 | Singleton | EMIEAVGYRSWPRYF | - | seq917 | EMIEAVGYRSWPRYF |
| 635.1 | Singleton | KQQGLNFSYLAAYQA | - | seq1068 | KQQGLNFSYLAAYQA |
| 636.1 | Singleton | SPAQDIWGTSAAAYF | - | seq1074 | SPAQDIWGTSAAAYF |
| 637.1 | Singleton | NTSSGASPPADASDS | - | seq715 | NTSSGASPPADASDS |
| 638.1 | Singleton | DKSKLQDVKDNKPSD | - | seq1121 | DKSKLQDVKDNKPSD |
| 639.1 | Singleton | GVTRPSAPPLPHVV | - | seq479 | GVTRPSAPPLPHVV |
| 640.1 | Singleton | LAPVPPPATRRRRAV | - | seq567 | LAPVPPPATRRRRAV |
| 641.1 | Singleton | RRQPIPKARRPE | - | seq643 | RRQPIPKARRPE |
| 642.1 | Singleton | APLTAVAPAHDTPPV | - | seq772 | APLTAVAPAHDTPPV |
| 643.1 | Singleton | QPAPATAPSQGGV | - | seq873 | QPAPATAPSQGGV |
| 644.1 | Singleton | IPPPPRGVPQIEVTF | - | seq930 | IPPPPRGVPQIEVTF |
| 645.1 | Singleton | GEIRPSAPPLPPVA | - | seq959 | GEIRPSAPPLPPVA |
| 646.1 | Singleton | GQSRGRGRGRGRGRGKG | - | seq1136 | GQSRGRGRGRGRGRGKG |
| 647.1 | Singleton | IALSQMGDA | - | seq826 | IALSQMGDA |
| 648.1 | Singleton | YNSSRSYWT | - | seq344 | YNSSRSYWT |
| 649.1 | Singleton | TMYPNRQPGSGWDSS | - | seq918 | TMYPNRQPGSGWDSS |
| 650.1 | Singleton | EFMKEADKEAY | - | seq346 | EFMKEADKEAY |
| 651.1 | Singleton | YVPSQERNFTTAPAI | - | seq907 | YVPSQERNFTTAPAI |
| 652.1 | Singleton | QPTVTLLPAADMDDF | - | seq349 | QPTVTLLPAADMDDF |
| 653.1 | Singleton | DAWREGEEFVVEFDL | - | seq358 | DAWREGEEFVVEFDL |
| 654.1 | Singleton | VGTTDRSGAPTY | - | seq373 | VGTTDRSGAPTY |
| 655.1 | Singleton | FWRGDLVFDFQV | - | seq763 | FWRGDLVFDFQV |
| 656.1 | Singleton | KAKASTVKAKLLSV | - | seq359 | KAKASTVKAKLLSV |
| 657.1 | Singleton | CLTFGRETVIEY | - | seq360 | CLTFGRETVIEY |
| 658.1 | Singleton | KIENAAMAIHLCFNN | - | seq361 | KIENAAMAIHLCFNN |
| 659.1 | Singleton | SKPTTKQRQNKPPSK | - | seq363 | SKPTTKQRQNKPPSK |
| 660.1 | Singleton | SKKPRQKRTATKQYNV | - | seq1003 | SKKPRQKRTATKQYNV |
| 661.1 | Singleton | QAFGRRGPEQTQGNF | - | seq682 | QAFGRRGPEQTQGNF |
| 662.1 | Singleton | RRKPKDEGAGVDKA | - | seq437 | RRKPKDEGAGVDKA |
| 663.1 | Singleton | KDDQIAAAMVLRGMA | - | seq1016 | KDDQIAAAMVLRGMA |
| 664.1 | Singleton | TYNWGENETDVFLL | - | seq440 | TYNWGENETDVFLL |
| 665.1 | Singleton | SGYDNDALYASV | - | seq375 | SGYDNDALYASV |
| 666.1 | Singleton | VVTVRAERPGVDPDR | - | seq380 | VVTVRAERPGVDPDR |
| 667.1 | Singleton | PGQGAYAAANSWVDV | - | seq818 | PGQGAYAAANSWVDV |
| 668.1 | Singleton | TEKFVKEQ | - | seq438 | TEKFVKEQ |
| 669.1 | Singleton | PGAPGGSGSGPRHRDGV | - | seq384 | PGAPGGSGSGPRHRDGV |
| 670.1 | Singleton | HDKNNSDI | - | seq1103 | HDKNNSDI |
| 671.1 | Singleton | NRRGLDLLFWEQGGL | - | seq389 | NRRGLDLLFWEQGGL |
| 672.1 | Singleton | KEEAEKKAAEQRALL | - | seq390 | KEEAEKKAAEQRALL |
| 673.1 | Singleton | ASGAKEEAEKKAAEQ | - | seq830 | ASGAKEEAEKKAAEQ |
| 674.1 | Singleton | VCAVDMPYLTVELIE | - | seq636 | VCAVDMPYLTVELIE |
| 675.1 | Singleton | GRFDEAMQDQIRQLE | - | seq997 | GRFDEAMQDQIRQLE |
| 676.1 | Singleton | GTVVAVGPGRWDEDG | - | seq508 | GTVVAVGPGRWDEDG |
| 677.1 | Singleton | DRSGAPTYSWGAND | - | seq635 | DRSGAPTYSWGAND |
| 678.1 | Singleton | NGGYLAAGKLTWADF | - | seq1045 | NGGYLAAGKLTWADF |
| 679.1 | Singleton | KIFPKKKEDNQAVDT | - | seq1051 | KIFPKKKEDNQAVDT |
| 680.1 | Singleton | ATNGDTHLGGEDFDN | - | seq1063 | ATNGDTHLGGEDFDN |
| 681.1 | Singleton | NSEDDDTFHDA | - | seq1104 | NSEDDDTFHDA |
| 682.1 | Singleton | SQVADKDDPTNKFY | - | seq1110 | SQVADKDDPTNKFY |
| 683.1 | Singleton | RELADALLE | - | seq960 | RELADALLE |
| 684.1 | Singleton | RRLGAALL | - | seq973 | RRLGAALL |
| 685.1 | Singleton | NYTYDSNYTP | - | seq596 | NYTYDSNYTP |
| 686.1 | Singleton | LADVPLDDDSDFVA | - | seq400 | LADVPLDDDSDFVA |
| 687.1 | Singleton | RIFYKDAFAKHQELF | - | seq809 | RIFYKDAFAKHQELF |
| 688.1 | Singleton | RGEHRDEHTQDAGDK | - | seq601 | RGEHRDEHTQDAGDK |
| 689.1 | Singleton | AEEKLKKKSSFYQS | - | seq411 | AEEKLKKKSSFYQS |
| 690.1 | Singleton | VATHLAGPQSSSAF | - | seq416 | VATHLAGPQSSSAF |
| 691.1 | Singleton | GLKAKPKFTLGKRKA | - | seq419 | GLKAKPKFTLGKRKA |
| 692.1 | Singleton | DQRPYCWHYP | - | seq1035 | DQRPYCWHYP |
| 693.1 | Singleton | QGTRRRYSSVQEPQA | - | seq449 | QGTRRRYSSVQEPQA |
| 694.1 | Singleton | SQVYTTSEYPSQ | - | seq425 | SQVYTTSEYPSQ |
| 695.1 | Singleton | DGVMSTRCPCGAAI | - | seq428 | DGVMSTRCPCGAAI |
| 696.1 | Singleton | WMNSTGFTKVCGAPPC | - | seq442 | WMNSTGFTKVCGAPPC |
| 697.1 | Singleton | KVMATIDSF | - | seq570 | KVMATIDSF |
| 698.1 | Singleton | SGTQVYQDPAIVQPK | - | seq589 | SGTQVYQDPAIVQPK |
| 699.1 | Singleton | VAKPADAVSTQSAKN | - | seq739 | VAKPADAVSTQSAKN |
| 700.1 | Singleton | DVENVDGLSLWEMYK | - | seq746 | DVENVDGLSLWEMYK |
| 701.1 | Singleton | AVSKPMGTQTHTMIF | - | seq774 | AVSKPMGTQTHTMIF |
| 702.1 | Singleton | ELYDTLKKVKKTV | - | seq778 | ELYDTLKKVKKTV |
| 703.1 | Singleton | SGKGQQQQGQTVTKK | - | seq798 | SGKGQQQQGQTVTKK |
| 704.1 | Singleton | GVTAACPHAGAKSFY | - | seq838 | GVTAACPHAGAKSFY |
| 705.1 | Singleton | NVYADSFVVKGDDVR | - | seq851 | NVYADSFVVKGDDVR |
| 706.1 | Singleton | YKRRMADAVSRKKM | - | seq865 | YKRRMADAVSRKKM |
| 707.1 | Singleton | SQTPRYRRPKFPWFK | - | seq1017 | SQTPRYRRPKFPWFK |
| 708.1 | Singleton | VYAWERKKISNCVAD | - | seq1139 | VYAWERKKISNCVAD |
| 709.1 | Singleton | RSQPRGRRQPIP | - | seq580 | RSQPRGRRQPIP |
| 710.1 | Singleton | AKAVYQQFYEFYEKV | - | seq754 | AKAVYQQFYEFYEKV |
| 711.1 | Singleton | WLQEGKGENKFV | - | seq784 | WLQEGKGENKFV |
| 712.1 | Singleton | EAEKQKAAEATKVA | - | seq813 | EAEKQKAAEATKVA |
| 713.1 | Singleton | DQVTKKEKKTIKKA | - | seq511 | DQVTKKEKKTIKKA |
| 714.1 | Singleton | TNDEEDTNDEEDTND | - | seq842 | TNDEEDTNDEEDTND |
| 715.1 | Singleton | GFTTNEERYNVFAE | - | seq919 | GFTTNEERYNVFAE |
| 716.1 | Singleton | GNRERRNTSDTVDDT | - | seq994 | GNRERRNTSDTVDDT |
| 717.1 | Singleton | ENDVLNQETEEEMEK | - | seq1086 | ENDVLNQETEEEMEK |
| 718.1 | Singleton | MRQLDTNVER | - | seq1089 | MRQLDTNVER |
| 719.1 | Singleton | IAAEQDRNTREVFAQ | - | seq1095 | IAAEQDRNTREVFAQ |
| 720.1 | Singleton | GVPIELQVECFTWDQ | - | seq463 | GVPIELQVECFTWDQ |
| 721.1 | Singleton | SLKLESSCNFDL | - | seq490 | SLKLESSCNFDL |
| 722.1 | Singleton | MKFREGSSEVC | - | seq539 | MKFREGSSEVC |
| 723.1 | Singleton | KTFPPTEPKKDKKKK | - | seq571 | KTFPPTEPKKDKKKK |
| 724.1 | Singleton | KDNTDEGDEGDDS | - | seq624 | KDNTDEGDEGDDS |
| 725.1 | Singleton | DAEKAVEVDPDDV | - | seq661 | DAEKAVEVDPDDV |
| 726.1 | Singleton | ADEEQQQALSSQMGF | - | seq812 | ADEEQQQALSSQMGF |
| 727.1 | Singleton | MAEMKTDAATLAQEA | - | seq1010 | MAEMKTDAATLAQEA |
| 728.1 | Singleton | GNGCFEFYHKCDNTC | - | seq1092 | GNGCFEFYHKCDNTC |
| 729.1 | Singleton | CSCLWMMLLISQAEA | - | seq450 | CSCLWMMLLISQAEA |
| 730.1 | Singleton | TQGCNCSIYP | - | seq453 | TQGCNCSIYP |
| 731.1 | Singleton | INEYTTGPSTPCPS | - | seq1038 | INEYTTGPSTPCPS |
| 732.1 | Singleton | EYDQVVVGAEYD | - | seq456 | EYDQVVVGAEYD |
| 733.1 | Singleton | LTCGFADLMGYIP | - | seq1044 | LTCGFADLMGYIP |
| 734.1 | Singleton | FPNITNLCPFGEVFN | - | seq465 | FPNITNLCPFGEVFN |
| 735.1 | Singleton | YDASVHGQIGAALL | - | seq1053 | YDASVHGQIGAALL |
| 736.1 | Singleton | DYVMNFNTVRYD | - | seq521 | DYVMNFNTVRYD |
| 737.1 | Singleton | AFDLDRLLFKRNICH | - | seq622 | AFDLDRLLFKRNICH |
| 738.1 | Singleton | YHYDADENSKQKKWD | - | seq743 | YHYDADENSKQKKWD |
| 739.1 | Singleton | IRLAKCGPWHRGGWD | - | seq614 | IRLAKCGPWHRGGWD |
| 740.1 | Singleton | MRRVAIAGVLAMRPE | - | seq733 | MRRVAIAGVLAMRPE |
| 741.1 | Singleton | ISMIDGPAPDGYPII | - | seq756 | ISMIDGPAPDGYPII |
| 742.1 | Singleton | SVPANVSRRAKVDVL | - | seq785 | SVPANVSRRAKVDVL |
| 743.1 | Singleton | YYALDVYAYDVT | - | seq506 | YYALDVYAYDVT |
| 744.1 | Singleton | KSQDADYH | - | seq1043 | KSQDADYH |
| 745.1 | Singleton | SRKKMDTKPTDPTG | - | seq1129 | SRKKMDTKPTDPTG |
| 746.1 | Singleton | AALVSGTATAGWTFG | - | seq1134 | AALVSGTATAGWTFG |
| 747.1 | Singleton | MPPYPTGGPPPV | - | seq523 | MPPYPTGGPPPV |
| 748.1 | Singleton | WPQIAQFAPSASAFF | - | seq537 | WPQIAQFAPSASAFF |
| 749.1 | Singleton | KDKKDDKYTKILAA | - | seq654 | KDKKDDKYTKILAA |
| 750.1 | Singleton | AFRQQFQYAVEV | - | seq664 | AFRQQFQYAVEV |
| 751.1 | Singleton | NMIVMKQLGEVRRFF | - | seq983 | NMIVMKQLGEVRRFF |
| 752.1 | Singleton | QIGYYRRATRRVRGG | - | seq1125 | QIGYYRRATRRVRGG |
| 753.1 | Singleton | LQQASKQAEAAAPVV | - | seq713 | LQQASKQAEAAAPVV |
| 754.1 | Singleton | VLQQLAVKTFGQPP | - | seq753 | VLQQLAVKTFGQPP |
| 755.1 | Singleton | AQQPSTATSVDAP | - | seq1034 | AQQPSTATSVDAP |
| 756.1 | Singleton | MLDLQPETTDLYCYE | - | seq569 | MLDLQPETTDLYCYE |
| 757.1 | Singleton | TGIEIVKRAI | - | seq911 | TGIEIVKRAI |
| 758.1 | Singleton | SSQRGRSGSGNF | - | seq573 | SSQRGRSGSGNF |
| 759.1 | Singleton | GASAQSGTSGTSGTS | - | seq792 | GASAQSGTSGTSGTS |
| 760.1 | Singleton | ALNDNAGRHNSE | - | seq991 | ALNDNAGRHNSE |
| 761.1 | Singleton | DNNDSGHSTGADTG | - | seq783 | DNNDSGHSTGADTG |
| 762.1 | Singleton | NCDVVIGIINNTVYD | - | seq819 | NCDVVIGIINNTVYD |
| 763.1 | Singleton | IGRLHLNDR | - | seq669 | IGRLHLNDR |
| 764.1 | Singleton | WNWFDITNW | - | seq637 | WNWFDITNW |
| 765.1 | Singleton | KLDDKDPQFKDNVILL | - | seq708 | KLDDKDPQFKDNVILL |
| 766.1 | Singleton | RGFLGRMLF | - | seq926 | RGFLGRMLF |
| 767.1 | Singleton | STCSEEDDSVVCCS | - | seq895 | STCSEEDDSVVCCS |
| 768.1 | Singleton | WIGNGYRY | - | seq1020 | WIGNGYRY |
| 769.1 | Singleton | KVIKKDVWNVISWVF | - | seq937 | KVIKKDVWNVISWVF |
| 770.1 | Singleton | AIANAYWSPQARRRF | - | seq995 | AIANAYWSPQARRRF |
| 771.1 | Singleton | RSFGSSSTSGITGD | - | seq1011 | RSFGSSSTSGITGD |
| 772.1 | Singleton | FHSTSSESN | - | seq663 | FHSTSSESN |
| 773.1 | Singleton | HHAERNGSQSTTSQSN | - | seq981 | HHAERNGSQSTTSQSN |
| 774.1 | Singleton | AKTAATNAEKKKTK | - | seq668 | AKTAATNAEKKKTK |
| 775.1 | Singleton | TGRPSHEAPNMTQ | - | seq738 | TGRPSHEAPNMTQ |
| 776.1 | Singleton | QVVYDYQH | - | seq698 | QVVYDYQH |
| 777.1 | Singleton | SYSEVGDVNVEE | - | seq986 | SYSEVGDVNVEE |
| 778.1 | Singleton | ALGLLQQASKQAEAA | - | seq773 | ALGLLQQASKQAEAA |
| 779.1 | Singleton | SGKDGHHAA | - | seq901 | SGKDGHHAA |
| 780.1 | Singleton | ASVASGASGGSGNSK | - | seq1115 | ASVASGASGGSGNSK |
| 781.1 | Singleton | FSKWRKNHMRQKSNK | - | seq712 | FSKWRKNHMRQKSNK |
| 782.1 | Singleton | ALYASVAVQQQD | - | seq1140 | ALYASVAVQQQD |
| 783.1 | Singleton | APMSTPSATSVR | - | seq737 | APMSTPSATSVR |
| 784.1 | Singleton | STPGSSRGNSPARMA | - | seq933 | STPGSSRGNSPARMA |
| 785.1 | Singleton | TSSTPPSGTEN | - | seq732 | TSSTPPSGTEN |
| 786.1 | Singleton | IGQMFETTMR | - | seq999 | IGQMFETTMR |
| 787.1 | Singleton | SVFHQNGQVTEV | - | seq927 | SVFHQNGQVTEV |
| 788.1 | Singleton | SRPSWGPTDPRR | - | seq1090 | SRPSWGPTDPRR |
| 789.1 | Singleton | IPSIQSRGLFGAIAG | - | seq1032 | IPSIQSRGLFGAIAG |
| 790.1 | Singleton | KAQKEATAAKLKA | - | seq899 | KAQKEATAAKLKA |
| 791.1 | Singleton | SRGGSQASSRSSSRSR | - | seq823 | SRGGSQASSRSSSRSR |
| 792.1 | Singleton | SRSSSRSRGNSRNST | - | seq1075 | SRSSSRSRGNSRNST |
| 793.1 | Singleton | GWTFGAGAALQIPFA | - | seq947 | GWTFGAGAALQIPFA |
| 794.1 | Singleton | GGSGGSVASGGSVAS | - | seq1022 | GGSGGSVASGGSVAS |
| 795.1 | Singleton | FVGAGLAGAAIGSV | - | seq1070 | FVGAGLAGAAIGSV |
| 796.1 | Singleton | DYEPPVVHGCPLPPP | - | seq955 | DYEPPVVHGCPLPPP |
| 797.1 | Singleton | SGPSGPSGTSPSSRS | - | seq949 | SGPSGPSGTSPSSRS |
| 798.1 | Singleton | TVAEEHVEEPTVAEE | - | seq958 | TVAEEHVEEPTVAEE |
| 799.1 | Singleton | QLTQAIVKNHKNLLK | - | seq974 | QLTQAIVKNHKNLLK |
| 800.1 | Singleton | LYGNEGCGWA | - | seq980 | LYGNEGCGWA |
| 801.1 | Singleton | AMPKTTYELKMECPH | - | seq1079 | AMPKTTYELKMECPH |
| 802.1 | Singleton | RCHARKAVAHINSV | - | seq1064 | RCHARKAVAHINSV |
| 803.1 | Singleton | WKTWGKAKM | - | seq1126 | WKTWGKAKM |
